# Research progress and prospect of key technologies of fruit target recognition for robotic fruit picking

**DOI:** 10.3389/fpls.2024.1423338

**Published:** 2024-12-06

**Authors:** Shaohua Liu, Jinlin Xue, Tianyu Zhang, Pengfei Lv, Huanhuan Qin, Tianxing Zhao

**Affiliations:** ^1^ College of Engineering, Nanjing Agricultural University, Nanjing, Jiangsu, China; ^2^ College of Artificial Intelligence, Nanjing Agricultural University, Nanjing, Jiangsu, China

**Keywords:** target recognition, fruit, machine vision, deep learning, robotic picking

## Abstract

It is crucial for robotic picking fruit to recognize fruit accurately in orchards, this paper reviews the applications and research results of target recognition in orchard fruit picking by using machine vision and emphasizes two methods of fruit recognition: the traditional digital image processing method and the target recognition method based on deep learning. Here, we outline the research achievements and progress of traditional digital image processing methods by the researchers aiming at different disturbance factors in orchards and summarize the shortcomings of traditional digital image processing methods. Then, we focus on the relevant contents of fruit target recognition methods based on deep learning, including the target recognition process, the preparation and classification of the dataset, and the research results of target recognition algorithms in classification, detection, segmentation, and compression acceleration of target recognition network models. Additionally, we summarize the shortcomings of current orchard fruit target recognition tasks from the perspectives of datasets, model applicability, universality of application scenarios, difficulty of recognition tasks, and stability of various algorithms, and look forward to the future development of orchard fruit target recognition.

## 1 Introduction

**Figure 1:**
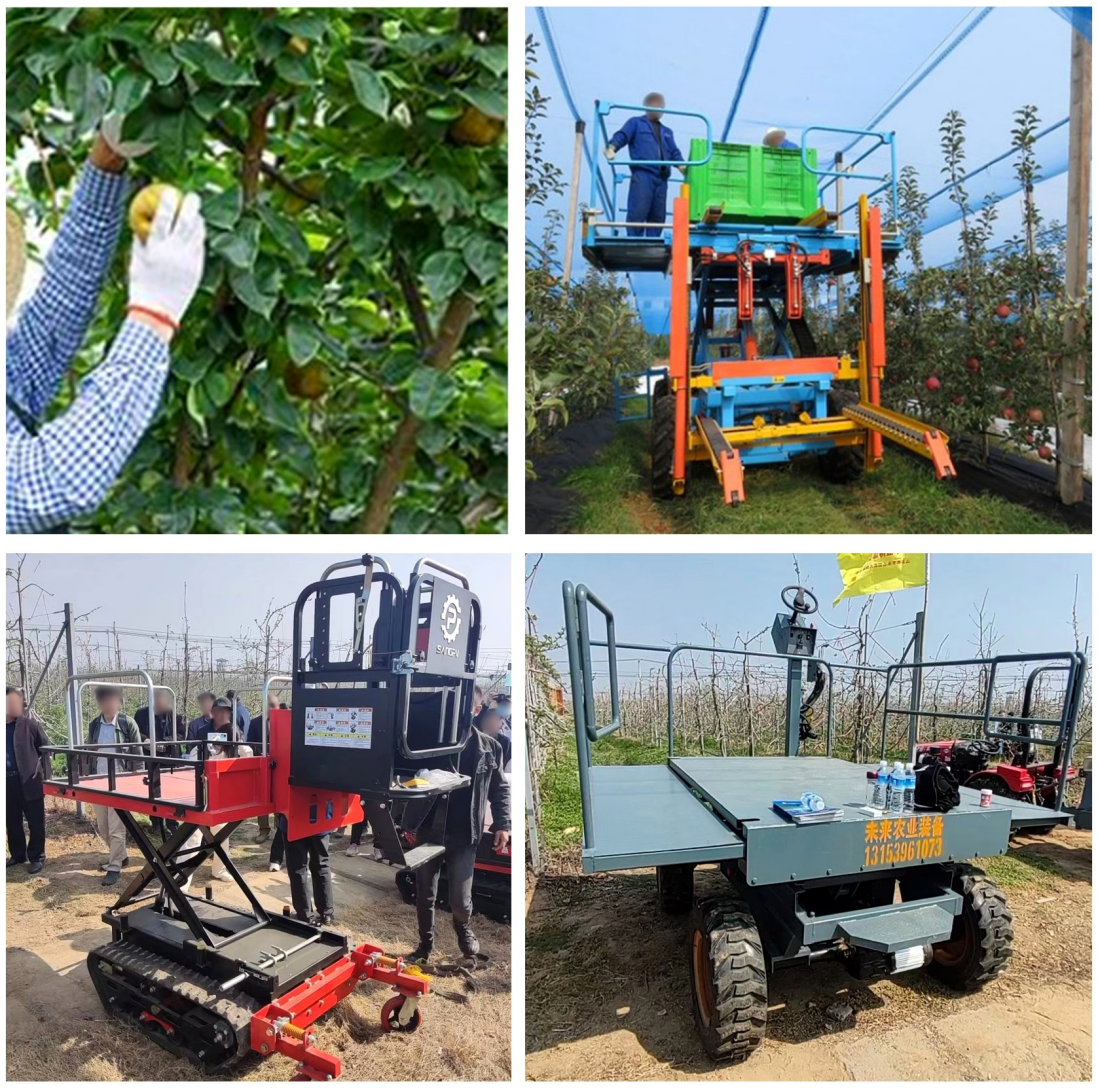
The main picking method in the market at this stage.

At present, manual picking is still used in most orchards, which have high labor intensity and low efficiency, making it difficult to guarantee picking technology and quality. With the rapid development of the fruit planting industry, the aging of the social population, and the transformation of the labor force’s employment concepts, the shortage of rural labor resources has become increasingly prominent, especially the demand for labor-intensive jobs such as fruit picking is also facing challenges. At present, the picking methods in the market mainly include manual picking and mechanically assisted semi-manual picking, as shown in **Figure 1**, which can no longer meet the market demand, a new picking method is needed to improve the efficiency and quality of fruit production.

Robotic fruit picking has been the focus of research recently and is also an important direction for the upgrading of the agricultural industry. Their widespread use in the facility agricultural production process can improve the production efficiency and quality of fruit picking and promote the sustainable development of the fruit industry. Machine vision is one of the key technologies for robotic fruit picking, which can be used to complete multiple functions such as fruit detection, recognition, and positioning (Bazame et al., 2021). This paper only introduces the relevant research on machine vision in fruit target recognition.

**Figure 2:**
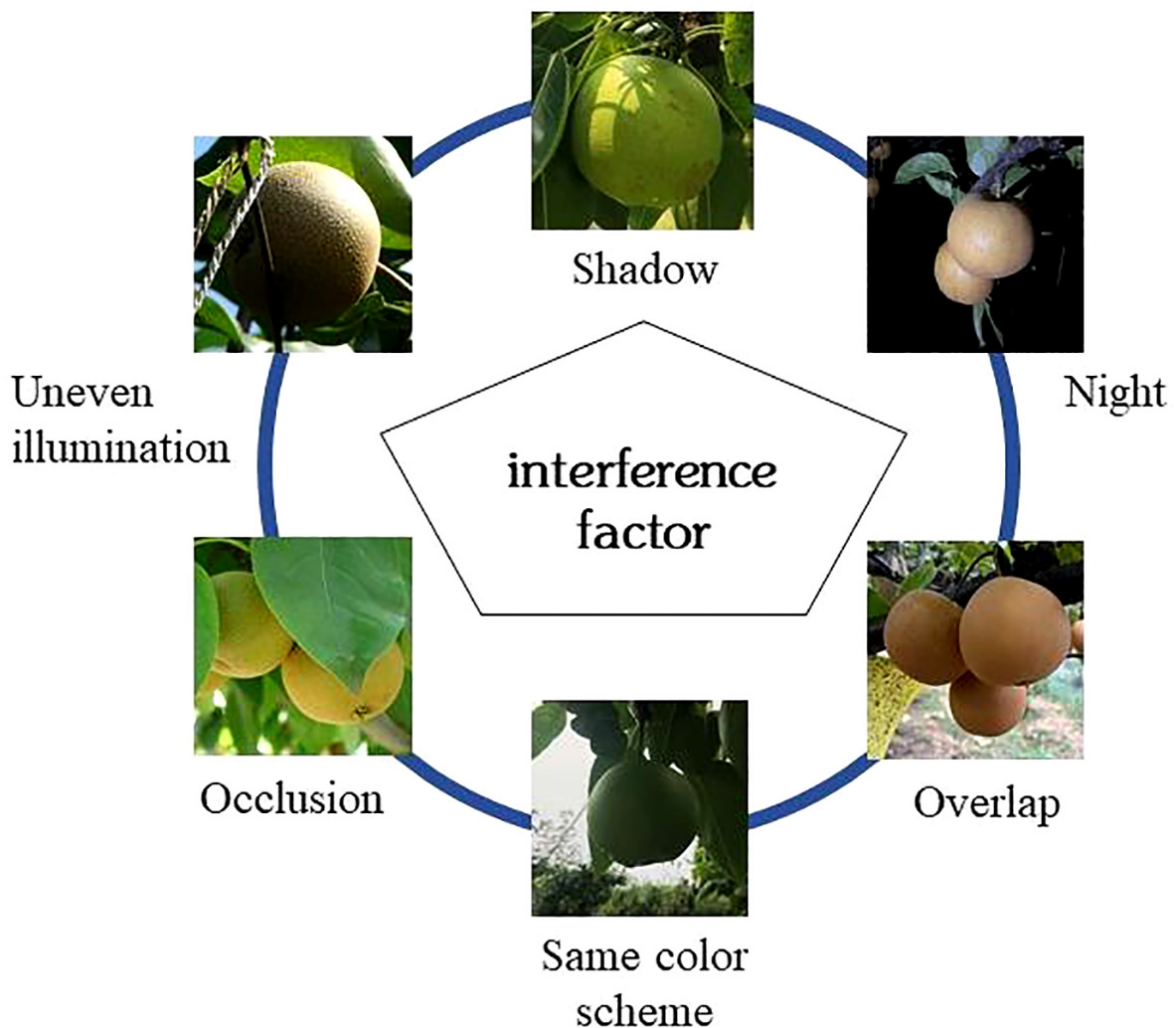
Orchard interference factors.

Due to the complexity and non-structured nature of the orchard’s environment, robotic picking still faces some challenges in fruit target recognition. Fruit target recognition methods can be divided into two categories: one is the traditional recognition method that artificially designs manual features based on the shape, color, and texture of the fruit itself, using algorithms such as chromatic aberration method, a threshold segmentation method, region growing method, support vector machine, and K-means clustering for image segmentation; another is the Convolutional Neural Networks(CNN) method based on deep learning (Chen et al., 2023). The detection algorithm of the traditional recognition method is relatively mature at present. However, in the complex environment of the natural orchard, due to the influence of factors such as shadows, uneven illumination, occlusion, night environment, fruit overlap, and the same color scheme, etc., as shown in **Figure 2**, making the traditional recognition methods manually designed features more complex (Cao et al., 2021; Tang et al., 2020), it is difficult to meet the operational requirements of actual fruit harvesting. The traditional detection algorithm mainly has shortcomings: low pertinence of the selection strategy, weak universality; large amount of calculation, slow detection speed and poor real-time performance; the low precision of recognition effect.

The CNN method based on deep learning has a high degree of hierarchical structure, has a strong selflearning ability for the features of the target, and can show a certain generalization ability, which makes this method have certain robustness when facing the complex environment of orchards, and also has a good performance in terms of detection accuracy and real-time performance. It is an end-to-end detection model that fuses feature extraction, selection, and classification of targets in the same model (Zhao et al., 2020; Huaibo et al., 2023). With powerful learning capability and highly hierarchical structure, it has unique advantages in fusing complex visual information with target perception (Li et al., 2022c). Although the CNN method based on deep learning has outstanding performance in fruit target recognition, there are still some problems in the complex environment of the orchard, and the maturity of the technology still cannot meet the requirements of practical operations.

**Figure 3:**
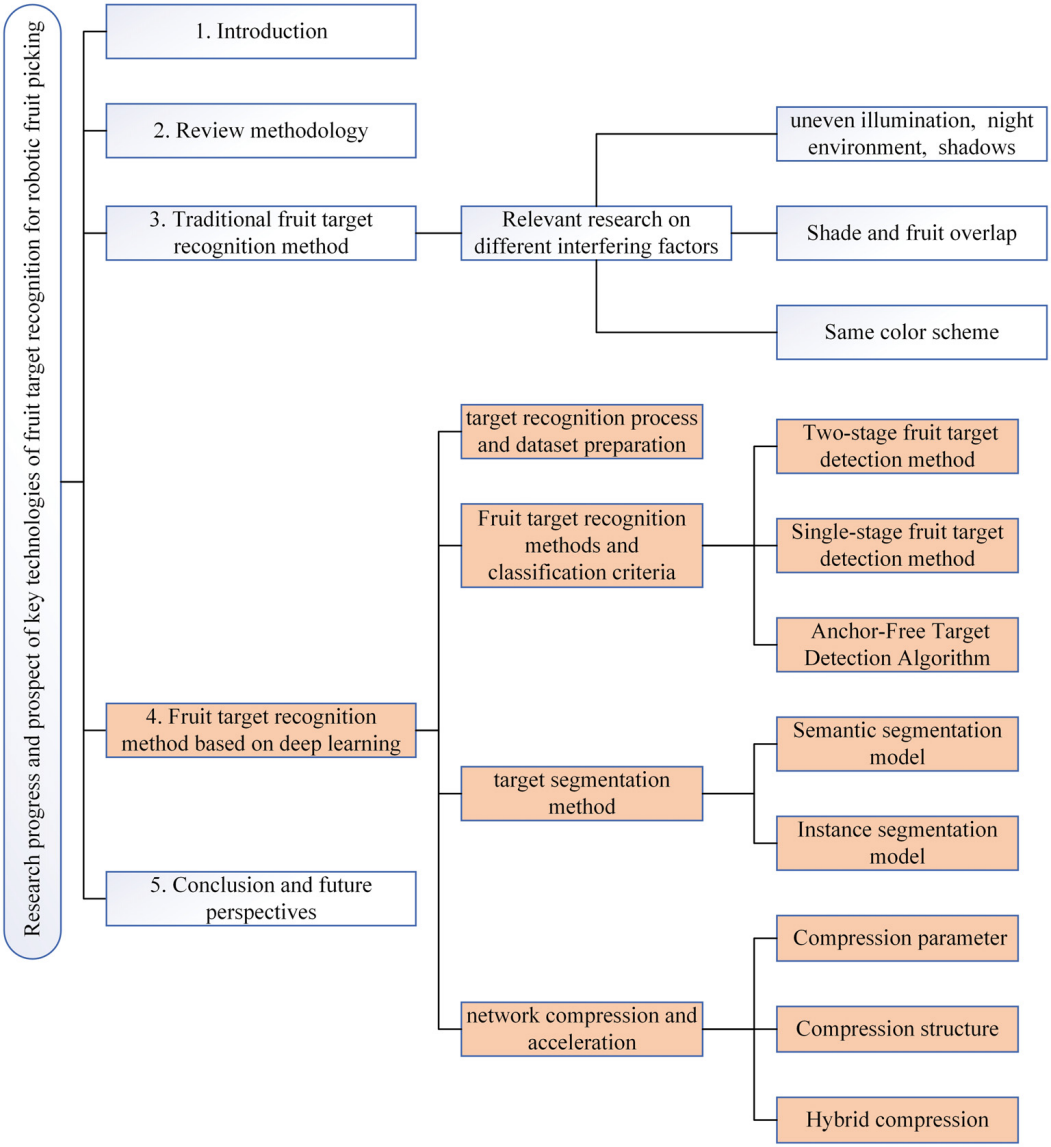
Content logic diagram.

This paper reviewed the research progress of fruit target recognition and high-quality articles related to key technologies, aiming to introduce the improvement and application of different recognition algorithms for fruit recognition, summarize the existing problems and challenges of fruit target recognition technology, and prospect the development direction of this technology. It can provide a reference for the research of fruit target recognition of robotic picking. In general, the traditional fruit target recognition method and fruit target recognition method based on deep learning were introduced, and the application of different recognition algorithms in fruit target recognition was summarized. Section 2 introduced the review methods involved in this paper, including the scope of literature retrieval, the databases and keywords used, and the visual results. Section 3 discussed the application of the traditional fruit target recognition method to different interference factors in the orchard. Section 4 is the key review part of this paper, which focuses on the fruit target recognition method based on deep learning. The fruit target recognition method based on deep learning was introduced in four parts: deep learning target recognition process and datasets preparation, fruit target recognition method and classification standard, target segmentation method, and fruit target recognition method based on network compression and acceleration. By comparing the research and application of different scholars in fruit target recognition algorithms, the advantages and disadvantages of different network models were summarized. Section 5 summarized and prospected the research trend of fruit target recognition based on deep learning. The logic diagram of the main review content in this paper as shown in **Figure 3**.

## 2 Review methodology

In this work, the methods of fruit target recognition and the research achievements and progress of related scholars in this field are reviewed, by searching relevant journal papers and conference papers in the past 18 years (2006-2023). The selected literature resources mainly come from the Web of Science database, in addition to multi-disciplinary databases (such as Elsevier ScienceDirect) and open online resources (such as open-access journals, academic websites, and academic forums). The keywords used to retrieve scientific and technological papers mainly include “deep learning”, “Machine vision”, “recognition”, “segmentation” and “lightweight”. The retrieval string in the Web of Science database based on the above keywords and Boolean search terms is (“recognition” OR “detection” OR “detect” OR “identify”) AND (“harvest” OR “harvesting” OR “pick” OR “picking”) AND (“fruit”) AND (“robot”) AND (“segmentation”) AND(“lightweight”). A total of 181 references were selected for review in this work, and 23 types of fruit target recognition research results were retrieved.

## 3 Traditional fruit target recognition method

With the development of deep learning, classification methods, detecting, and segmenting fruit targets based on manual features are defined as the traditional target recognition methods. Compared with the deep learning target recognition algorithms, the traditional target detection algorithm has certain limitations, which are only suitable for recognition scenarios with simple detection backgrounds and obvious target features, such as apples, peaches, and other fruits with obvious differences in color from leaves. By distinguishing the pixel color difference between the target and the background region based on the color features, the target fruit can be separated from the background. To realize the recognition of lychee fruits and fruit stems, Xiong Juntao et al. used the YCbCr color model to perform threshold segmentation on lychee images based on the color and grayscale features of lychees (Xiong et al., 2011). Si Yongsheng et al. used normalized red-green differential segmentation to segment apples and backgrounds based on color features and achieved the recognition of red apples (Si et al., 2010). To recognize immature tomatoes, Ma Cuihua et al. conducted relevant research based on significance detection and improved circular random Hough transformation, with a correct recognition rate of 77.6% (Ma et al., 2016). However, in the field environment in the actual natural background, there are many interference factors for the recognition of fruit targets, so it is difficult to realize accurate recognition of fruits through general abstract features. To overcome the influence of field interference factors on accurate recognition, some scholars have conducted the following related research on different interference factors.

Given interference factors such as uneven illumination, night environment, and shadows, some scholars have improved the lighting conditions during image acquisition, such as using a light-blocking device to block the strong light when the scene light is strong and providing an auxiliary light source to optimize the lighting conditions when the light is weak. FAN et al. considered the influence of lighting and shadows, a pixel block segmentation method based on gray-centered red, green, and blue (RGB) color space was proposed to effectively distinguish apple fruit pixels from other pixels by exploring the color characteristics and local changes of apple images (Fan et al., 2021b, a). TSOULIAS et al. used LiDAR to target changes in lighting conditions and proposed an apple detection method based on corrected backscattering reflection intensity (R-ToF) and geometric features, which could alleviate the influence of lighting changes on fruit recognition (Tsoulias et al., 2020). GONGAL et al. aimed at the halo and shadow interference factors on the fruit surface built an opaque tunnel structure, and installed auxiliary light sources to weaken the influence of canopy occlusion and illumination changes on fruit recognition. Based on multi-features and patches, an apple image segmentation technique was proposed by using grey-centered red, green, and blue color space (Gongal et al., 2016). In response to shadow interference factors, Zhao De’an et al. adopted the method of auxiliary light source to increase the incandescent lamps at different angles to weaken the shadow on the fruit (Dean et al., 2015). For the nighttime environment, JIA et al. used different auxiliary light sources such as incandescent lamps, fluorescent lamps, and LED lamps to collect images by filling light processing for nighttime apple images, and concluded that the color feature images of incandescent lamps were more similar to those of natural light images through comparative analysis (Jia et al., 2018). To overcome the influence of natural light on image segmentation, Lv et al. used an adaptive gamma correction method to obtain a complete and clean fruit area (Lv et al., 2019b). Lv et al. also designed a green apple image segmentation method that combines the normal bright areas and the highlight areas of the fruit (Lv et al., 2019a). To eliminate shadows produced under strong illumination and direct sunlight conditions, Xu et al. combined group pixels and edge probability graphs to develop a new algorithm with strong robustness for the detection of orchard apples under natural illumination conditions (Xu et al., 2019). Based on super-pixel features, Liu Xiaoyang et al. proposed a fruit segmentation method for apple-picking robots for the recognition and segmentation of unevenly colored fruits in the natural environment, which is better than the chromatic aberration method using pixel-level features and the segmentation method using neighborhood pixel features and meets the real-time demands (Xiaoyang et al., 2019). Based on the observation of highlight points under artificial illumination, LINKER et al. proposed a new method for detecting apples in nighttime images by analyzing the spatial distribution of light around highlights (“bright spots”) (Linker and Kelman, 2015).

In the non-structured orchard environment where fruits are blocked by branches and leaves, fruits overlap with each other, and the combination of overlapping fruits and branches and leaves has a serious impact on fruit recognition. JIA et al. extracted a total of 16 features such as fruit color and shape based on a pulse-coupled neural network, introduced a Genetic algorithm(GA) to optimize the Elman neural network, and proposed a new genetic Elman neural network (GA-Elman), with a recognition rate of 88.67% for overlapping fruits (Jia et al., 2020a). Color and illumination factors have a great impact on traditional target recognition algorithms. To address this problem, Liu Changyuan et al. proposed a fruit recognition and localization algorithm based on depth images from the perspective of fruit morphology, which can effectively deal with the overlapping and occlusion scenes of fruits, and realize the picking work at night (Liu et al., 2022a). Regarding the problem of overlapping tomatoes, Xiang Rong et al. realized the recognition of overlapping fruits based on edge curvature analysis, but with the increase of the occlusion rate, the recognition precision would decrease significantly (Xiang et al., 2012). TAO et al. proposed an automatic apple recognition method based on point cloud data to process apple image information. Based on color fusion (extraction of RGB and HSI color components) and three-dimensional geometric information (FPFH), targets were divided into fruits, branches, and leaves (Tao and Zhou, 2017). NYARKO et al. proposed a new RGB-D image method of fruit recognition based on convex surface detection and classification for fruit recognition in leaves and branches, aiming at the condition of occluded and Shadowed fruits (Nyarko et al., 2018).

Fruits with epidermal similarity to branches and leaves, such as Cuiguan pear, Su Cui pear, green lemon, citrus, etc., are called same color scheme fruits. For such fruits, a single color feature cannot distinguish them, so it is necessary to combine color, shape, texture, and other multi-feature recognition. Regarding the problem of homochromatic citrus, KURTULMUS et al. proposed a new “feature fruit” detection method based on color and circular Gabor texture analysis (Kurtulmus et al., 2011). SUN et al. proposed a progressive detection method for green apples based on fuzzy set theory to enhance the image and (AIM) algorithm to determine the fruit region, to achieve accurate segmentation of fruit targets (Sun et al., 2020). LI et al. used significance detection and a Gaussian curve fitting algorithm to represent the image as a closed loop graph with super-pixels as nodes, then sorted the nodes and finally binarized them to detect green apples in natural scenes (Li et al., 2018). It is difficult to recognize green apples in a natural light environment, Liao Wei et al. established a green apple random forest recognition model, carried out Otsu threshold segmentation and filtering processing based on RGB color space, extracted the grayscale and texture features of leaves and apples, realizing the classification and recognition of green apple fruits in this type of environment (Wei et al., 2017). SUN et al. designed a GrabCut model based on a visual attention mechanism to solve the same color scheme problem. For overlapping fruits, the Ncut algorithm was used to accurately segment the extracted fruits (Sun et al., 2019).

Many scholars have conducted in-depth research on the interference factors of fruit recognition in nonstructured orchards and proposed corresponding recognition methods for fruits in each specific scene. However, with the continuous improvement of people’s requirements for orchard-picking technology, traditional image processing methods have been unable to meet the needs of picking robots, and it is difficult to popularize the traditional recognition methods in practical applications. The main reason is that the traditional hand-design features (color, texture, and shape) become more complex due to uncertainty interference factors, and the limited artificial features can not meet the needs of fruit picking in a variety of scenarios, resulting in the traditional image processing methods are limited, which can not adapt to the real-time and universal nature of fruit harvesting operations in the orchard. The main defects are as follows: (1) Traditional image processing methods have more redundant regions in the candidate regions, low utilization rate, large algorithm model, and complex feature extraction process, resulting in increased computation and slow detection speed; (2) Artificial features cannot adapt to multiple picking conditions under complex background, feature descriptors designed based on low-level visual cues are only suitable for simple scenes, and it is difficult to extract representative semantic information for recognition tasks under complex background. (3) The hand-designed features for specific fruits have great limitations, poor classifier self-adaptation, and weak generalization ability, making it difficult to generalize the application to other fruits.

## 4 Fruit target recognition method based on deep learning

With the advancement of artificial intelligence technology, deep learning has made significant progress in recent years. The architecture of deep learning models is constantly evolving, and the feedforward neural network is the original deep learning model architecture. With the continuous development of deep learning, CNN, recurrent neural network (RNN), Transformer, and so on gradually appear. Evolving deep learning benefits from the availability of large-scale datasets and increasingly powerful computing power. This data can be used to train more accurate models, and advances in high-performance computing hardware (e.g., GPUs and TPUs), have made it possible to train deeper and more complex models. Compared with the traditional recognition direction, the target recognition method based on deep learning has the advantages of self-learning of target features, strong expression ability, good generalization performance, high recognition precision and real-time performance, a large number of scholars have begun to apply it to fruit target recognition.

The fruit target recognition methods based on deep learning typically use CNN (LeCun et al., 2015), introducing multi-layer perceptrons in the structure, and using low-level features to form high-level features. With multilayered representation, it can learn non-structured features under different interference factors from training datasets through machine learning and has higher precision and universality for fruit target recognition. These methods train the network with a large number of labeled fruit images so that it can learn the features of different fruits. At the time of recognition, the model extracts features from the input images and compares them with the trained data to determine the type of fruit in the images. Common deep learning frameworks such as TensorFlow and PyTorch can be used to implement these methods.

Based on the recognition results of detection components and target regions, deep learning models can be divided into classification and detection models (image classification and target detection) and segmentation models (semantic segmentation and instance segmentation), which are also the four basic tasks of machine vision. Since the source code of the deep learning model is mostly open source for researchers to use, the vast majority of scholars who do fruit recognition are based on the characteristics of the target fruit itself and the growing environment of the orchard to improve the research based on better network models for visual recognition (such as R - CNN, YOLO, etc.), to achieve the goal of faster recognition speed and precision of fruit recognition under complex orchard environment, to meet the requirements of picking.

### 4.1 Deep learning target recognition process and dataset preparation

The specific steps of fruit target recognition based on deep learning (based on better model improvement) include dataset preparation, target detector selection, model structure modification, modified model transfer training, model application testing and evaluation, and model continuous improvement. Among them, the preparation of datasets is a key step in deep learning, and also the basis of deep learning target recognition tasks. The preparation of datasets includes image acquisition, data cleaning, data labeling, data segmentation, data enhancement, and other steps. The quality and diversity of the datasets will affect the final training results and recognition precision of the model. Therefore, for the non-structured orchard environment, the amount of image acquisition data must be large enough, and fruit images under various interference factors in the complex orchard environment should be included as much as possible. However, due to the periodic harvesting of fruits and the non-structural nature of the orchard itself, as well as the influence of weather, region, fruit species, time, human, and other factors, the current preparation process of orchard datasets is complicated, time-consuming and laborious, for the recognition research in this field has not yet a representative orchard public datasets for researchers to use.

The deep learning training process can be specifically divided into supervised learning, unsupervised learning, semi-supervised learning, and weakly supervised learning according to whether the data has label information.

Supervised Learning: In supervised learning, the training datasets contain inputs and corresponding labels (or outputs). The model learns these data to create a mapping of inputs to outputs that allow it to predict new and unseen data. Classical classification and regression tasks fall into the supervised learning category (Caruana and Niculescu-Mizil, 2006). It is also the current main method of fruit target recognition based on deep learning. The datasets preparation has a great impact on supervised learning, and the richness of data information in the training data directly affects the final recognition effect, the size of the datasets is usually determined by the deep learning model and image complexity. For the fruit recognition task under the complexity of non-structured orchards, the datasets should contain multiple types of image data in the orchard complex environment under various interference factors, such as shadow, branch occlusion, fruit overlap, night environment, uneven illumination, and the same color scheme, and the data scale should be large enough. Since fruits are cyclical ripening crops, weather, time, region and other factors make it difficult to prepare orchard datasets, which increases the difficulty of the picking work.

Unsupervised Learning: In unsupervised learning, the training data has no corresponding label and only contains input. The goal of the model is to discover the intrinsic structures, patterns, or features in the data, such as tasks such as clustering (grouping data into groups) and dimensionality reduction (reducing the dimensions of the data). Unsupervised learning omits the more complex process of data labeling, and with original data samples model can extract distinguishable information or features from the structure of training data, and then map the features extracted from the input image to the specified output (Hasan et al., 2021).

Semi-Supervised Learning: Semi-supervised learning is a learning mode between supervised and unsupervised learning, in which the label coverage of the training datasets is not all image data, but only part of the image data is labeled. This method uses the powerful self-learning ability of deep learning to map the relationship between labeled data and unlabeled data and improve the detection performance of the model. Semi-supervised learning can use as much information as possible to achieve better generalization capabilities when the data is limited or the cost of label production is high so that training with small and medium-sized data can obtain high-precision results (Xiao et al., 2020b).

Weakly Supervised Learning: A training model in which there is only partial label information in the training data is called weakly supervised learning. This information may be rough and incomplete labels. In this case, the model needs to learn about the data from the incomplete label information for tasks such as target detection, segmentation, etc. Incomplete supervision, Inexact supervision, and Inaccurate supervision are three typical types of weakly supervised learning (Zhou, 2018). This is a growing field, and researchers are constantly coming up with new ways to improve the effectiveness of weakly supervised learning.

### 4.2 Fruit target recognition methods and classification criteria based on deep learning

The rapid development of Deep Learning began in 2012 when AlexNet overwhelmingly defeated traditional target detection algorithms in the ImageNet Large-scale Visual Recognition Challenge (ILSVRC) (Krizhevsky et al., 2017). In 2013, the European Commission and Baidu respectively initiated and established the supercomputer project and the Deep Learning Research Institute. In 2014, two influential CNN models, VGGNet and Inception Net (GoogLeNet), were developed. Then deep learning developed more and more rapidly, in the development of algorithms related to object recognition as shown in **Table 1**. Algorithms not indicated with references in the table are network models published on platforms such as GitHub.

**Table 1:** Major development history of object recognition algorithm based on deep learning.

Year	Development stages of recognition algorithms
2012	AlexNet(early CNN) (Krizhevsky et al., 2012)
2013	OverFeat (Sermanet, 2013), ZFNet (Zeiler, 2014)
2014	VGG (Simonyan and Zisserman, 2014), GoogLeNet (Szegedy et al., 2015), R - CNN (Girshick et al., 2014)
2015	SPPNet (He et al., 2015) and ResNet (He et al., 2016) (Multi-scale Feature Extraction and Deeper Network Layers), Faster R - CNN (Girshick, 2015), YOLO (Redmon et al., 2016) (single-stage object detection algorithms are beginning to emerge)
2016	SDD (Liu et al., 2016), YOLOv2 (Redmon and Farhadi, 2017)
2017	The model begins to integrate tasks such as instance segmentation, semantic segmentation, and object detection, Mask R - CNN (He et al., 2017a) introduces the concept of instance segmentation, MobileNet (Howard, 2017), ShuffleNet(efficient model) (Zhang et al., 2018)
2018	YOLOv3, CornerNet (Law and Deng, 2018)
2019	ExtremeNet (Zhou et al., 2019), FCOS (Tian et al., 2019), CenterNet (Duan et al., 2019), FoveaBox (Kong et al., 2020), EfficientNet (Tan and Le, 2019) (efficient model), GhostNet (Han et al., 2020), CondConv (Yang et al., 2019)
2020	YOLOv4 (Bochkovskiy et al., 2020), YOLOv5, RegNet (Radosavovic et al., 2020) (Efficient model)
2021	YOLOF (Chen et al., 2021a), YOLOR (Wang et al., 2021a), YOLOX (Zheng et al., 2021)
2022	YOLOv6 (Li et al., 2022a), YOLOv7 (Wang et al., 2023b)
2023	YOLOv8

The object recognition detection algorithm based on deep learning can be divided into two categories: classification-based two-stage detection algorithm and regression-based single-stage detection algorithm. Two-stage detection algorithms divide the target detection problem into two stages: first, the candidate target frames are generated, and then these frames are classified and positionally adjusted. This method typically requires two forward passes. Representative algorithms include R - CNN, Fast R - CNN, Faster R - CNN, and Mask R - CNN, among others. It is characterized by accurate detection results, high detection precision, and wide adaptability to the target size. The single-stage detection algorithm treats the object detection problem as a regression problem and only needs one forward pass to predict both the category and boundary frame of the object at the same time. It is a method that can predict the target location and classification directly from the image. YOLO (You Only Look Once) (Redmon et al., 2016) and SSD (Single Shot MultiBox Detector) (Liu et al., 2016) are two typical single-stage detection algorithms. It is characterized by simple and fast, multi-scale prediction, relatively less calculation, and better performance for small and dense targets.

The choice of a single-stage or two-stage detection algorithm depends on the application scenario, computing resources, and requirements for detection performance. In general, the single-stage algorithms have the advantage in terms of speed and are suitable for real-time or fast detection requirements, while the two-stage algorithms perform better in terms of precision and are suitable for tasks that require high precision.

#### 4.2.1 Two-stage fruit target detection method

**Figure 4:**
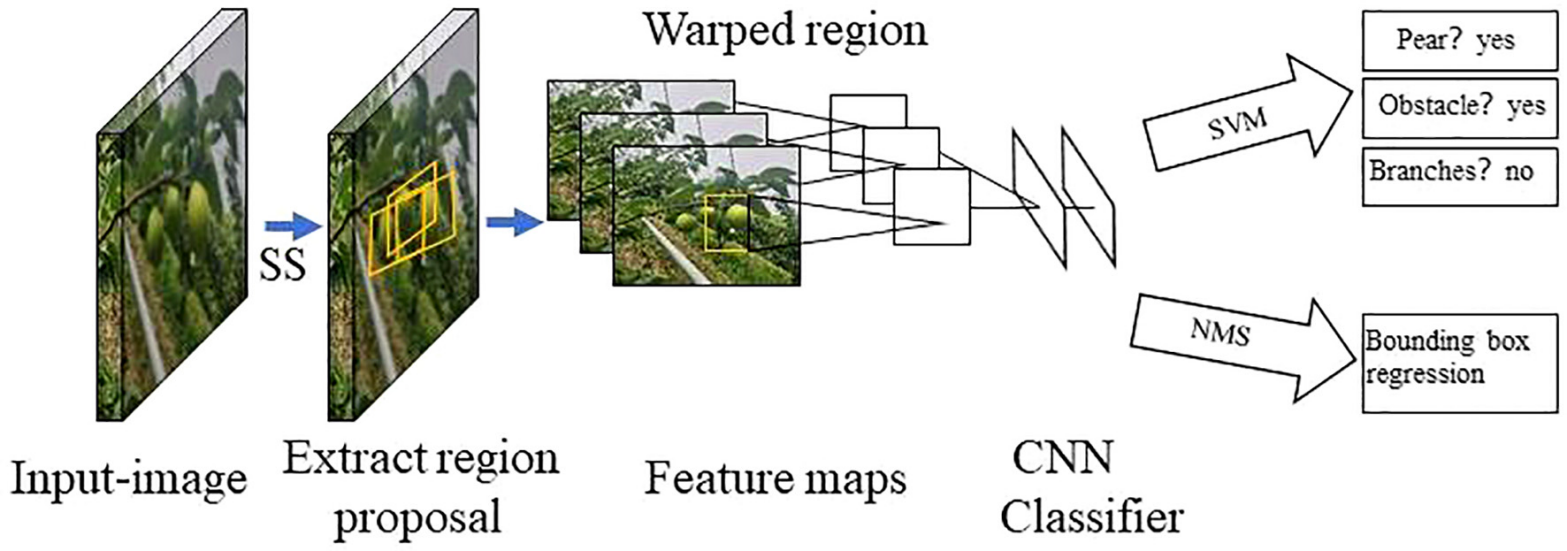
R - CNN network model structure diagram.

**Figure 5:**
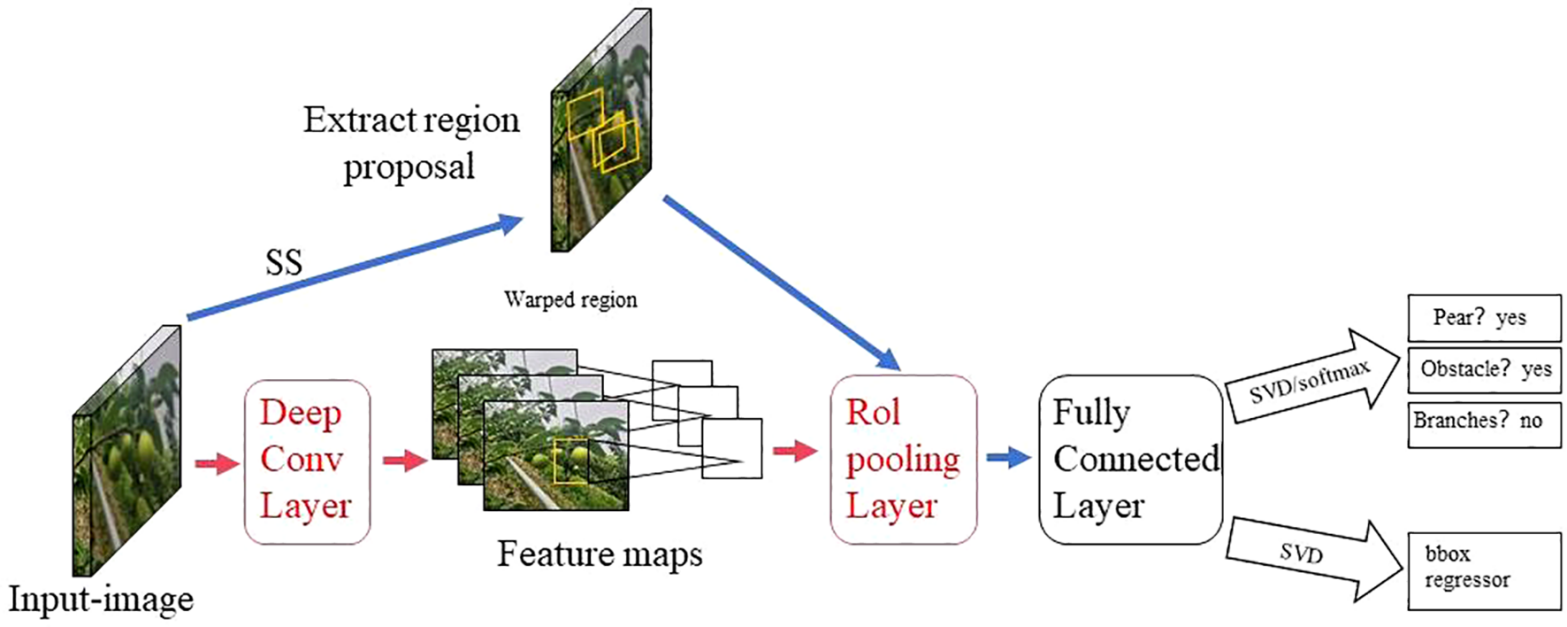
Fast R - CNN network model structure diagram.

**Figure 6:**
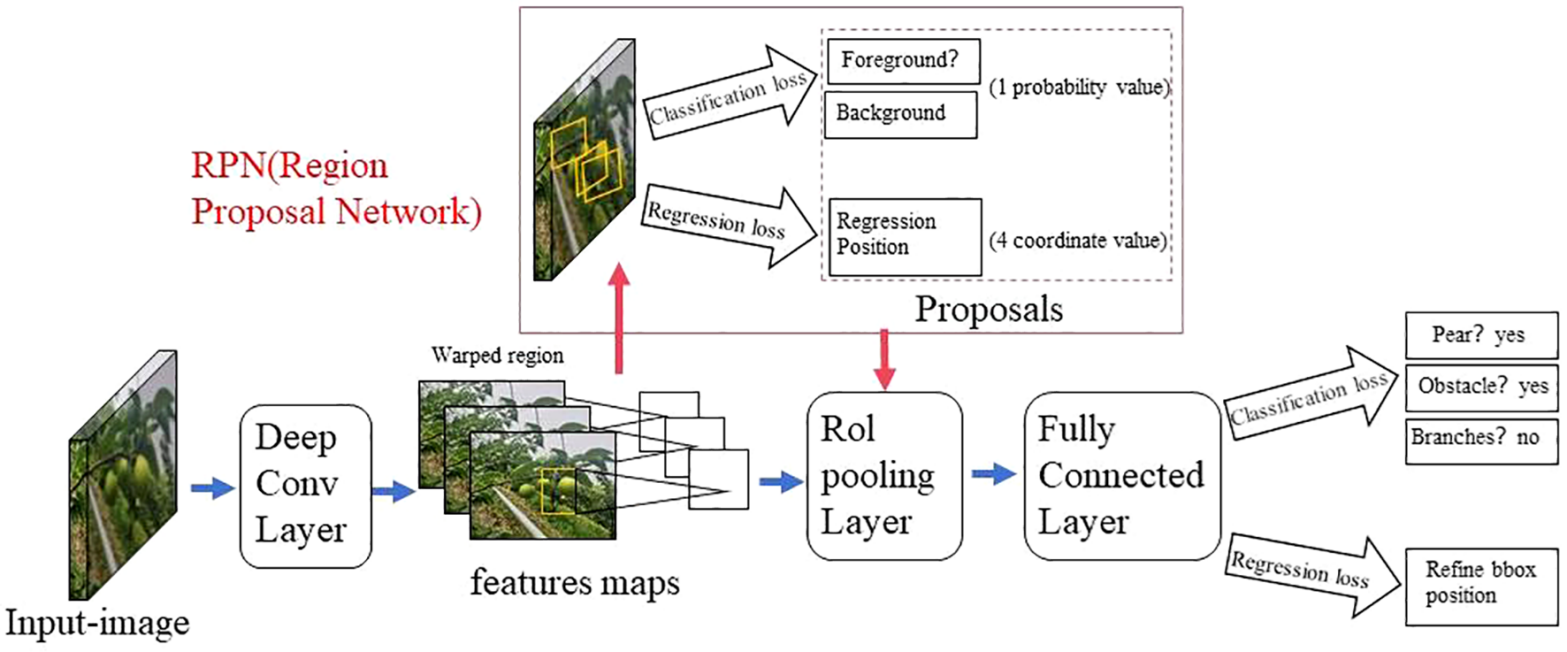
Faster R - CNN network model structure diagram.

**Figure 7:**
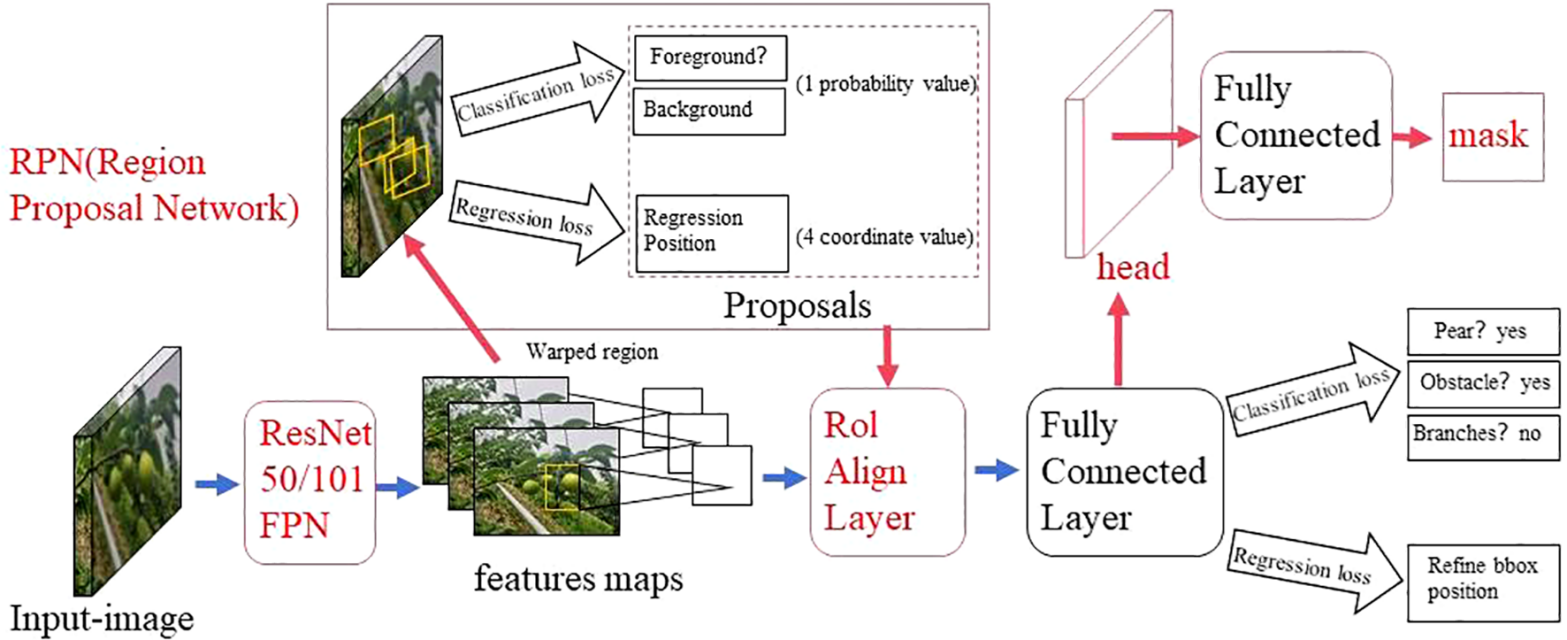
Mask R - CNN network model structure diagram.

The two-stage detection algorithm is not a simple fusion of traditional machine learning methods and CNN, but rather a specific target detection method, which uses CNN based on deep learning for target detection, but adopts a two-stage process in the target detection process. Candidate frame generation stage: In this stage, the algorithm generates a series of candidate target frames through different methods, often referred to as “candidate regions” or “candidate frames”. These candidate frames are regions that may contain targets, but their category and precise location have not yet been determined. Target classification and position adjustment stage: In this stage, the generated candidate frames are passed through the CNN for target classification (i.e., determining which category they belong to) and position localization (i.e., adjusting the coordinates of the bounding box). This stage uses deep learning methods, usually using CNN to achieve classification and localization. This is different from traditional machine learning methods in algorithmic ideas and processes. Girshick et al. proposed the R - CNN algorithm inspired by the AlexNet network (Girshick et al., 2014), The network structure is shown in **Figure 4**. The training and testing of the network take a long time, occupy a large space, and the training modules are independent of each other. Fast R - CNN (Girshick, 2015) adds an RoI pooling based on the previous R – CNN, then integrates the entire model using a deep convolutional neural network for efficient target detection, reducing the calculation area and increasing the training speed by 9 times, but the memory consumption is relatively large. The network structure is shown in **Figure 5**. The Region Proposal Network (RPN) is a highlight of Faster R-CNN, which replaces the SS (Selective Search) method to extract proposals. The network structure is shown in **Figure 6**. This structure greatly improves the speed of generating candidate regions for network models (Ren et al., 2015). Based on Faster R - CNN, Mask R - CNN adds a branch of segmentation task to predict the target mask, and fuses object detection and image segmentation into the same network. The network structure is shown in **Figure 7**, which uses a ResNet-FPN network with stronger feature extraction capability. To solve the problem of misalignment between RoI and extracted features, the RoI Align layer is introduced while the extracted features are aligned with the input (He et al., 2017b).

A large number of scholars have used the two-stage algorithms to accomplish the task of fruit target recognition under complex backgrounds in orchards. The relevant research results of the two-stage fruit target detection algorithm are shown in **Table 2**. As can be seen from **Table 2**, the two-stage algorithms such as Faster R - CNN and Mask R - CNN are applied to fruit target recognition in different scenarios, which can achieve higher detection precision and better performance in small target detection. However, the extracted feature maps are all single-layer with lower resolution. For occluded targets, the recognition precision will decrease. Moreover, the algorithm structure finally uses a fully connected layer, which occupies a large part of the parameters and increases the amount of calculation. The overall time of detection and segmentation is relatively long, and the detection speed is significantly slower than that of the single-stage detection algorithm. The next section will focus on the application of the single-stage detection algorithm.

**Table 2:** Research results of fruit target recognition based on a two-stage algorithm.

Recognition algorithm	Application scenarios	Technical principles and characteristics	Identification effect and evaluation index	Research scholars
Faster R - CNN	winter jujube Same-color, green walnut	Based on data balance, combined with deep learning Improved Faster R -CNN: Adding batch normalization and improving the adaptability	Improved generalization effect, mAP was 98.5% The precision was 97.71%, recall was 94.58%, F1 value was 96.12%, detection speed was 227 ms	(Wang et al., 2020; Fan et al., 2021c)
	Shake grab, apple	Improved Faster R - CNN: Transfer learning using pretrained networks such as Alexnet, VGG16	The mAP was 82.40% with an average detection time of 0.45 s	(Zhang et al., 2020)
	Light and Occlusion, passion fruit	Improved Faster R - CNN: Based on Multi-scale Fast Regional CNN	The precision was 93.10%, recall was 96.20%, F1 value was 94.60%,	(Tu et al., 2020)
	Same color, green citrus	Determine the optimal training parameters for the model	The mAP was 85.49% with an average running time of 0.4 s	(Juntao et al., 2018)
	Occlusion, fruit overlapping, kiwi	Transfer learning, using Im-AlexNet as a feature extraction layer	Complex environment, the mAP was 96%, and the detection speed was 1 s/graph	(Longtao et al., 2019)
	Natural environment prickly pear	Improved Faster R - CNN: Bilinear interpolation was used to change the ROI pooling to ROI align	The recall was 96.93%, precision was 95.53%, F1 was 94.99%, average speed was 0.2s/graph	(Yan et al., 2019)
	Multiple types of fruit	Improved Faster R - CNN framework: Improved convolution layer and pooling layer	The mAP was 92.51% and the speed was 58 ms/graph	(Wan and Goudos, 2020)
	Occlusion, apple	A multi-class apple detection method based on Faster R - CNN was proposed using VGG16	The average mAP of the four types of scenarios 87.9%	(Gao et al., 2020)
Mask R - CNN	Overlap fruit, apple	The input parameters are reduced, and each fruit the mask can be output	The precision rate was 97.31% and the recall rate was 95.70%	(Jia et al., 2020b)
	Block, overlap fruit, apple	Proposed RS-Net. Mask R - CNN was extended by embedding the Gaussian attention module	The mAP 86.2 with an average segmentation time of 65.79ms	(Jia et al., 2022b)
	Light, occlusion, apple	Add a suppress branch to standard Mask R - CNN to suppress non-apple features produced by the original network	The precision was 88.0%, recall was 93.10%, F1 value was 90.5%, detection time was 0.25 s/graph	(Chu et al., 2021)
	Shade, overlap, occlusion, apple	Improved Mask R - CNN: Added attention module (deformable convolution combined with deformable attention with key content items)	The precision was 95.8%, recall was 97.1%, F1 value was 96.4%, mAP was 91.7%	(Wang and He, 2022)
R - FCN	Same color, green apple	Improved R - FCN image feature extraction based on ResNet-44	The recall was 85.7%, precision was 95.1%, error rate was 4.9%, average speed was 0.187s/graph	(Wang and He, 2019)

#### 4.2.2 Single-stage fruit target detection method

**Figure 8:**
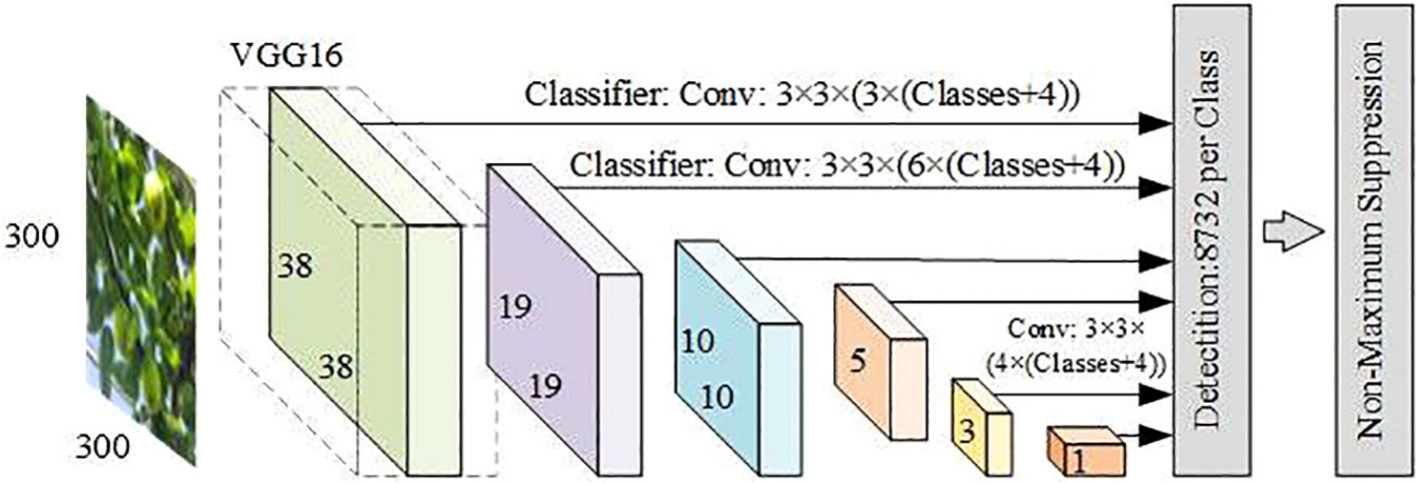
SSD network model structure diagram.

The single-stage target detection algorithms are also known as regression-based detection methods. This regression-based method enables the single-stage algorithm to complete the target location and classification in a single forward pass, with faster detection speeds compared with the two-stage detection algorithms. The YOLO series and SSD are two representative algorithms among them. SSD was proposed by Wei Liu et al. in 2016 (Liu et al., 2016). It can complete both target classification and location in a model at the same time and can adapt to multi-scale targets, which is fast and suitable for real-time target detection, but there is the problem of inaccurate location when the target scene is more complex, the network structure is shown in **Figure 8**.

YOLO target detection algorithm is an early single-stage target detection algorithm of deep learning. It was proposed by REDMON et al. in 2015 and is also a popular target detection algorithm at present (Redmon et al., 2016). Its core idea is that through a single CNN structure directly from image input to the final prediction result, including the generation of candidate boxes, target classification, and the prediction of boundary box regression parameters, it has already been derived from several generations of models. The latest detection model is YOLOv9 launched in 2024. **Table 3** lists some of the fruit target recognition research results based on a single-stage target detection algorithm. **Table 3** mainly includes two algorithms: SSD and YOLO. SSD is comparable to YOLO in terms of running speed, and comparable to the two-stage detection algorithm Faster R - CNN in terms of detection precision, but the setting process of min size, max size, and aspect ratio in the prior box needs to be completed manually. making the parameter debugging process more complicated and relying on manual experience. Therefore, YOLO is the single-stage algorithm with the highest usage rate and the most improvement at present, and this paper only analyzes the mainstream version officially released by YOLO.

**Table 3:** Research results of fruit target recognition based on a single-stage algorithm.

Recognition algorithm	Application scenarios	Technical principles and characteristics	Identification effect and evaluation index	Research scholars
SSD	The drone image is small in size, litchi	MFEFF-SSD model based on multiple feature enhancement and feature fusion was proposed. Add RFB, multi-scale feature fusion, attention mechanism	The average precision of a small target detection was higher than other classical models compared	(Peng et al., 2022)
	Lingwu Long Jujube	Improve the DenseNet model, introduce the Inception module, and feature fusion structure. Lightweight model	The mAP was 96.60%, the speed was 28.05 frames/s, number of parameters was 1.99 × 10	(Wang and Xue, 2021)
	Small target missed, misdetected, grapefruit	Design features Fusion single lens detector IFSSD, backbone network Inceptionv3, and Focal Loss function.	The precision rate was 93.70%, and the detection time was 29 s/graph	(Xiao et al., 2020a)
	Multiple types of fruit	Propose SD(single shot multibox detector) model and replace VGG16 with Res Net-101	The mAP was 89.53%, F1 value was 96.12%	(Peng et al., 2018)
YOLO	Tomato	Multi-scale IMS - YOLO algorithm, backbone Darknet-20, fuses multi-scale information	The mAP was 97.13%, recision was 96.36%, recall rate was 96.03%, the detection time was 7.72 ms	(Liu et al., 2020a)
YOLOv2	Same color, mango	Using YOLOv2 to realize green mango recognition in UAV image	The average detection time was 0.08 s/image and the AP was 86.43%	(Xiong et al., 2018)
	Light, same color, unripe mango	Tiny - Yolo network structure was designed to realize multilayer feature reuse and fusion	The detection speed was 83f/s, precision rate was 97.02%, recall rate was 95.1%	(Xue et al., 2018)
	strawberries	Image enhancement algorithm based on YOLOv3 combined with gamma transform	The mAP was 87.51%, precision was 97.14%, recall rate was 94.46%, detection rate was 58.1f/s	(Liu et al., 2020b)
YOLOv3	Night environment, litchi	Detection of litchi fruit at night was realized based on YOLOv3 and U-Net	AP value High brightness was 96.78%, normal brightness was 99.57%, and low brightness was 89.30%	(Liang et al., 2020)
	Light, occlusion, overlap, winter jujube	The improved YOLOv3-SE model was proposed, and the SE Block structure was introduced to enhance the feature expression ability of the feature map	Percentage improvement: recall rate was 2.43\∼5.08, mAP was 2.38\∼4.81, F1 value was 1.75\∼2.77	(Tianzhen et al., 2021)
	Night environment, overlapping citrus	Multi-scale CNN DesYOLOv3 algorithm, adding Dense Block structure	The mAP was 90.75% (Improved by 2.27%), detection speed increased by 11 f/s	(Xiong et al., 2020)
	Light, overlapping, occlusion, Apple	Fusion of DarkNet53 and CSPNet, adding SPP module to achieve feature fusion, using Soft NMS algorithm and joint Loss function based on Focal and CIoU Loss	The mAP 96.30%, F1 value 91.80%, detection speed 27.8 f/s	(Zhao et al., 2021)
	Pineapple	Based on binocular stereo vision and improved YOLO v3 model. DenseNet and SPP modules were added to the network	F1 and AP were 93.00% and 97.55% respectively in the slightly obscured datasets	(Liu et al., 2023)
	Complex background, Apple	Lightweight Light-YOLOv3 model, residual blocks in series, using depthseparable convolution, the multiobjective loss function is proposed	The detection speed and precision were improved, F1 value of 94.57%, mAP value of 94.69%	(Xing et al., 2020)
	Light, block, stick, bagging, apple	An apple recognition and location method based on YOLOv3 CNN was proposed	The mAP was 87.71%, precision was 97%, recall rate was 90%, IOU was 83.61%	(Zhao et al., 2019)
	Same color, light, Shade, banana	Multi-class detection of banana bunches and banana stalks based on YOLOv4	The model mAP was 93.69%, average detection time was 44.96 ms/graph	(Fu et al., 2022)
YOLOv4	Light, occlusion, tomato	YOLOv4 combines HSV to segment the target	When the segmentation area proportion was 16%, the precision was 94.77%, and the detection speed was 25.86ms/graph	(Li et al., 2021c)
	Light, occlusion, interference, tomato	To improve the backbone network, the deep separable convolution model is adopted to realize the reuse of feature information and multi-scale fusion	The precision rate was 88.00%, the recall rate was 89.00%, the mAP was 94.44%, detection speed was 10.71 f/s	(Zheng et al., 2022b)
	Small targets, strawberries	proposed, which adopts a lightweight network GhostNet to embed attention mechanism and integrate multi-features	The weight of the model was 4.68MB, the detection time was 5.63 ms/graph, and mAP was 92.62%	(Sun et al., 2022)
	Small target, dense, occluded, citrus	The feature recursive fusion network model FR-YOLOv4 was proposed, and the backbone network uses CSPResNest50 and RFP fusion features	The mAP is 94.60%, average detection speed 51 f/s	(Yi et al., 2021)
	Small target, same color, apple	The YOLOv4-SENL model was proposed, and two attention mechanisms, SE block, and NL block, were used to integrate advanced features.	With an average precision of 96.90%, the detection effect was better than SSD, YOLOv4, Faster R - CNN, and other models	(Song et al., 2021)
	Bright light, blurred image, occlusion, apple	The YOLOv4-NLAM-CBAM model was proposed, and two attention modules NLAM and CBAM were added	The AP of highlight/shadow, blurry and severely occluded images were 98.00%, 96.20% and 97.00%, respectively	(Jiang et al., 2022)
	Occlusion, size, apple	Improved model CAYOLOv4 was proposed, and CBAM convolutional attention module was added, adaptive layer and dense connection were introduced	The precision rates of early, middle, and harvest were 86.20%, 87.50%, and 92.60%, respectively	(Lu et al., 2022)
	Uneven lighting, occlusion, blueberries	The I-YOLOv4-Tiny network was proposed, CSPDarknet53Tiny was adopted as the backbone network, and the CBAM module was added	The mAP was 96.24%, the average detection time was 5.72 ms, and the memory occupied by the network structure was 24.20 MB	(Wang et al., 2021b)
	Small target, occlusion, tomato	A YOLOv4-tiny-X model was proposed, and CBAM was added, the Mish activation function was adopted, and global feature fusion was enhanced with DCCN	The detection speed on Nvidia GTX 2060 and global feature fusion was enhanced with DCCN	(Yang et al., 2022b)
	Small targets, strawberries	Propose a lightweight RTSDNet network, reduce the number of CSPNet modules, and simplify the network structure of CSPNet	Compared with the YOLOv4-Tiny model, mAP reduces by 0.62%, but the detection speed increases by 25.93%	(Zhang et al., 2022b)
	Citrus	Based on YOLOv5s combined with an improved visual significance detection algorithm	The mAP was 95.40%, occupying 13.70 MB of memory, detection time was 70 ms/graph	(Chen et al., 2022)
YOLOv5	Night environment, tomato	The CIoU target position loss function based on crossover ratio was used to calculate and select the best anchor frame size	The average recognition precision of tomatoes was 96.80%	(He et al., 2022)
	Natural environment, cherry	Adopt offline and online data enhancement strategies, add Transformer module, BiFPN structure, and P2 module	The precision rate was 97.60%, the recall rate was 89.90%, mAP was 95.20%	(Zhang et al., 2022c)
	Occlusion, overlap, apple	Improved YOLOv5s improves the bottleneck CSP module to bottleneck CSP-2, introduces the attention mechanism SE, improves the initial anchor frame size	The recall rate was 91.48%, precision was 83.83%, mAP was 86.75%, F1 value was 87.49%, speed was 15 ms/graph	(Yan et al., 2021)
	Stem occlusion, apple	Design BottleneckCSP module, introduce SE module, improve the initial anchor frame size of the network	The recall rate was 85.90%, precision was 81.00%, mAP was 80.70%, F1 value was 83.40%, speed was 25 ms/graph	(Bin et al., 2022)
	Grapes	An MRWYOLOv5s grape detection model was proposed. MobileNetv3 was used to extract features and attention mechanism, and RepVGG Block was introduced	Parameters size is 7.56M, mAP was 97.74% (2.32% higher), detection time was 10.03ms/graph (6.13ms lower)	(Sun J. et al., 2023)
	pear	The YOLO - P model was proposed. SB structure and ISB module were used to replace CBS structure, CBAM module was inserted, activation function: Hard-Swish	The AP was 97.6% (1.8% improved), model size was 8.3MB (39.4% compression), detection precision was 97.6%	(Sun H. et al., 2023)
	Elevated cultivation, strawberries	The ATCSP-YOLOv5s model is proposed and an attention mechanism is introduced. The effective segmentation of fruit stems was realized	The precision was 97.24%, recall rate was 94.07%, average precision was 95.59%, detection speed was 17.3f/s	(Yang et al., 2023)
	Growth type, apple	The YOLOv5-B network model with BiFPN-s structure was proposed, activation function: ACON-C	The average precision is 98.45% and the processing speed is 71 FPS	(Lv et al., 2022)
YOLOv7	Multigrowth posture, dragon fruit	Based on the optimal YOLOv7 model, a multi-pose dragon fruit detection method was proposed	The precision rate was 83.6%, the recall rate was 79.9%, and the mAP was 88.3%	(Wang et al., 2023)
	Fruit thinning period, apple	Merge the window-long selfattention mechanism, add Swin Transformer Block and adopt SIoU loss function	The average precision was 95.2%, precision was 92.7%, recall rate was 91.0%, model size was 81 MB	(Long et al., 2023)
	Different ripening, occlusion, tomato	MobileNetV3 was used to extract features and the global attention mechanism GAM was introduced	The precision rate was 98.6%, recall rate was 98.1%, mAP was 98.2%, detection time was 82ms	(Miao et al., 2023)
	Light, dragon fruit	The RDE-YOLOv7 detection method was proposed, introducing RepGhost and decoupling head and several ECA blocks	The precision, recall, and mAP were increased by 5.0%, 2.1%, and 1.6% respectively	(Zhou et al., 2023b)
	Complex orchard environment, pineapple	Insert the attention mechanism SimAM, improve the MPConv structure, and replace the nonmaximum inhibition (NMS) algorithm with a soft NMS algorithm	The mAP was 95.82% (2.71% improved), recall was 89.83% (3.41% improved)	(Lai et al., 2023)
	immaturity, occlusion, yellow peach	The YOLOv7-peach model was proposed, the CA module was embedded, EIoU was adopted, P2 shallow downsampling module was added	The mAP was improved by 3.5%, and the detection speed was up to 21 fps	(Liu and Yin, 2023)
	High density, occlusion, overlap, Apple	Introducing MobileOne module, improving SPPCSPS module to parallel channel, adding auxiliary detection head	precision improved by 6.9%, recall improved by 10%, mAP1 improved by 5%, mAP2 improved by 3.8%	(Yang et al., 2023b)
	Shade, small target, Apple	Lightweight YOLOv7-tiny algorithm, adding jump connection on shallow features used P2BiFPN for multi-scale feature fusion and reuse	The mAP was 80.4% (5.5% improved), loss rate was 3.16%	(Ma et al., 2023)
YOLOv8	Tomato	Depth-separable convolution, DPAG module is designed, and feature enhancement module is added	The mAP was 93.4% (1.5% improved), precision was 2% better, recall was 0.8% better	(Yang et al., 2023a)

For the first time, YOLO proposes a real-time end-to-end target detection method that uses a more direct output to predict detection outputs based solely on regression. The YOLOv1 structure consists of 24 convolutional layers followed by two fully connected layers for predicting the coordinates and probabilities of the bounding boxes. The network layer uses leaky RELU, and only the last layer uses linear activation functions and a 1 × 1 convolution layer to reduce the number of feature maps and keep the number of parameters relatively low. YOLOv1 unifies the target detection step by simultaneously detecting all bounding boxes and achieved an average precision(AP) of 63.4%on the PASCAL VOC2007 datasets, which had larger location errors than the Fast R - CNN of the same period.

The YOLOv2 has several improvements over the original YOLO to make it better, maintain the same speed, and be more powerful - capable of detecting 9,000 classes. The main improvements are as follows: 1. Batch normalization processes all convolutional layers in the network. 2. A high-resolution classifier of 448 × 448 is used to fine-tune the model. 3. Dense layers are removed and a fully convolutional architecture is used. 4. A pooling layer is removed and a pass-through layer is used to generate finer-grained features. 5.YOLOv2 does not use the full connection layer, and the input can be multi-scale images. With all these improvements, YOLOv2 achieved an average precision of 78.6% on the PASCAL VOC2007 dataset.

The YOLOv3 backbone network is Darknet-53, which replaces all maximum pooling layers with stride convolution and adds residual connections. It contains a total of 53 convolutional layers. The main improvements are as follows: 1. In terms of boundary box prediction, YOLOv3 uses logistic regression to predict an object property score for each boundary box. 2. In terms of class prediction, binary cross entropy is used to train independent logical classifiers, and the problem is formalized into multi-label classification. 3. YOLOv3 predicts three boxes on three different scales for multi-scale prediction. This helps to get a finer detail box and significantly improves the prediction for small objects, which was one of the main weaknesses of previous versions of YOLO. Since that release, all YOLO models have been evaluated in the MS COCO datasets, and the YOLOv3-spp has achieved 36.2% AP and 60.6% AP50 at 20 FPS, reaching the state-of-the-art level at the time, and the speed was increased by 2 times. At this point, the structure of the target detector begins to be divided into three parts: the backbone network, the neck network, and the head network. The backbone network is responsible for extracting useful features from the input images. The neck is the intermediate component that connects the backbone network to the head, focusing on enhancing spatial and semantic information at different scales. The head is the final component of the target detector, which makes predictions based on the features provided by the backbone network and the neck.

The main change in YOLOv4 is the enhanced architecture integrated with methods that slightly increase the cost of inference but significantly improve precision. The best-performing architecture is a modification of Darknet-53, adding a cross-stage partial connection (CSPNet) and a Mish activation function as the backbone network, and the neck network uses a modified path aggregation network (PANet) and a modified space Attention Module (SAM). CIoU loss and Cross mini-batch Normalization (CmBN) were added to collect statistics from the entire batch rather than from a single mini-batch and perform hyperparameter optimization with a genetic algorithm. Evaluated on test-dev 2017 on the MS COCO datasets, YOLOv4 achieved 43.5% AP and 65.7% AP50 at over 50 FPS on the NVIDIA V100.

The YOLOv5 introduces the Focus module and SPP structure, as well as the CSP module and FPN- PAN structure, to improve the efficiency of feature extraction and fusion, backbone network adopts CSPDarknet53, starting with Stem, that is, a stride convolution layer with large window size, SPPF (Spatial pyramid pool fast) layer and subsequent convolution layer to process features at different scales, while the upper sampling layer increases the resolution of the feature map. Each convolution is followed by batch normalization (BN) and SiLU activation. The neck uses SPPF and modified CSP-PAN, while the head is similar to YOLOv3. YOLOv5 uses multiple enhancement techniques, such as Mosaic, copy-paste, random affine, MixUp, HSV enhancement, random horizontal flipping, and other enhancements from the albumentations package, to increase the diversity of data; As evaluated on the MS COCO datasets test-dev 2017, YOLOv5x achieved 43.5% AP and 65.7% AP50 at 640 pixels image size at speeds over 50 FPS, using NVIDIA V100.

The YOLOv6 uses the RepVGG-based backbone network EfficientRep, which has higher parallelism than the previous YOLO backbone. The neck uses PAN, which is enhanced by RepBlocks or CSPStackRep modules, and for larger models, the highly efficient decoupled head after YOLOX is used. Classification VariFocal losses and SIoU/GIoU regression losses are used. Use RepOptimizer and channel-level distillation for faster detectors. Evaluated on test-dev 2017 of the MS COCO datasets, the largest model reached 57.2% AP at about 29 FPS on an NVIDIA Tesla T4.

Architectural changes in The proposed Extended Efficient Layer Aggregation Network (E-ELAN) and a new connection-based model scaling strategy are the structural highlights of YOLOv7. Evaluated on the MS COCO datasets test-dev 2017, YOLOv7-E6 achieved 55.9% AP and 73.5% AP50 at an input size of 1280 pixels at 50 frames per second, using an NVIDIA V100.

**Figure 9:**
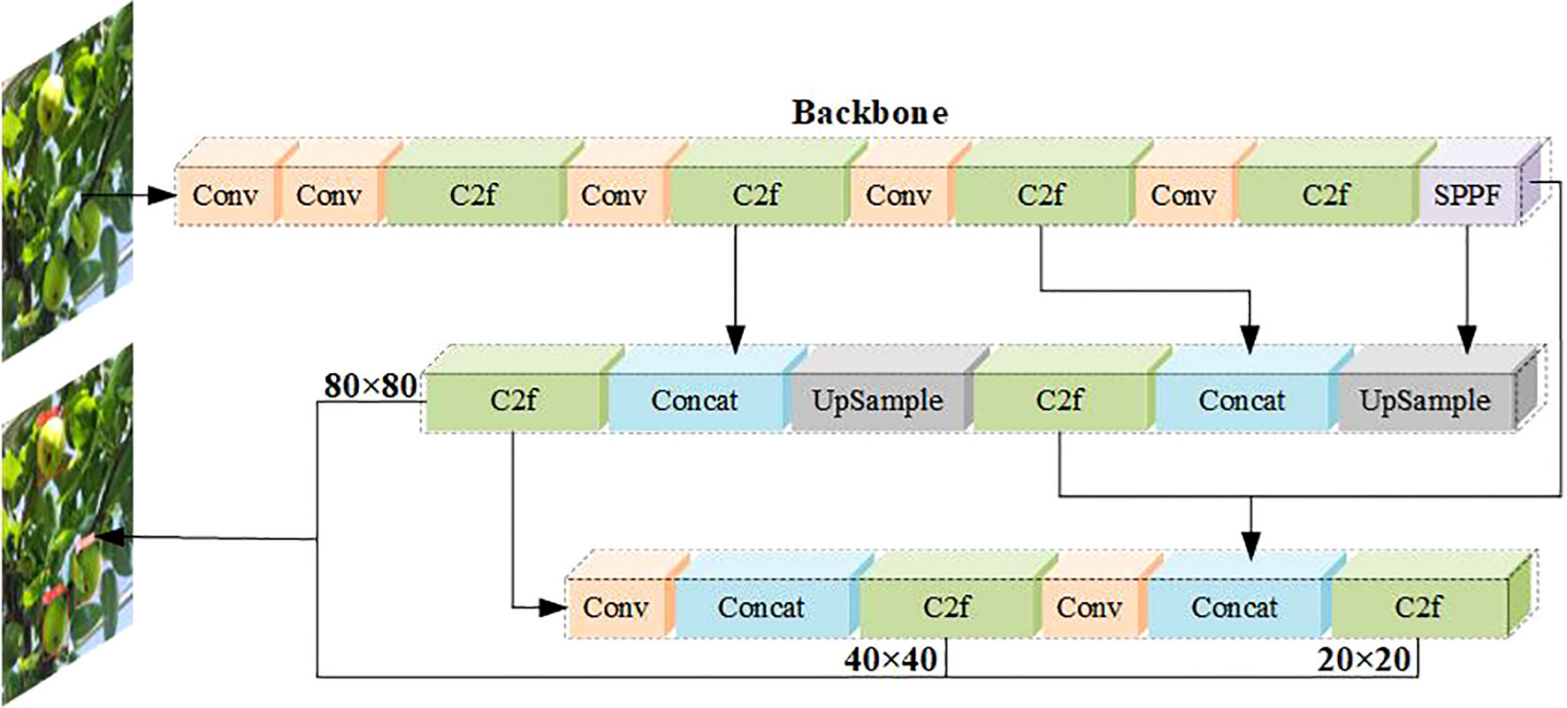
YOLOv8 network model structure diagram.

The YOLOv8 uses a backbone network similar to YOLOv5, with some modifications to CSPLayer, reducing the number of blocks of the maximum stage in the backbone network, thereby reducing the number of parameters and calculations, and achieving lightweight, called the C2f module. The convolution structure of the up-sampling phase on PAN-FPN is also optimized to combine high-level features with contextual information to improve detection speed and accuracy. It uses an anchor-free model with decoupling heads that independently handle object properties, classification, and regression tasks. The Sigmoid function is used as the activation function of the object property score, the Softmax function is used to represent the class probability, the CIoU and DFL loss functions are used to calculate the bounding box loss, and the binary cross entropy loss is used to calculate the classification loss. A semantic segmentation model named YOLOv8-Seg is provided whose backbone network is the CSPDarknet53 feature extraction, followed by a C2f module instead of the traditional YOLO neck architecture, the C2f module is followed by two segmentation heads that learn to predict semantic segmentation masks for input images. The YOLOv8 consists of five detection modules and a prediction layer, the model structure is shown in **Figure 9**. The YOLOv8-Seg model achieves state-of-the-art results on a variety of object detection and semantic segmentation benchmarks while maintaining high speed and efficiency. Evaluated on the MS COCO datasets test-dev 2017, The YOLOv8x achieved 53.9% AP at an image size of 640 pixels (compared to 50.7% for YOLOv5 at the same input size), running at 280 FPS on the NVIDIA A100 and TensorRT.

The YOLOv9 was released in February 2024. The main improvement is to propose programmable gradient information (PGI) and design GELAN, a new lightweight network architecture based on gradient path planning, which reduces parameters and calculation requirements. Compared with YOLOv8x, the parameters are reduced by 15%, reducing the calculation amount, but the AP value is increased by 1.7%. Since it has just been released, this article will not introduce the structure, readers can refer to the YOLOv8 structure for understanding the YOLO structure.

Many versions of YOLO have evolved around the idea of balancing speed and precision, providing real-time performance without sacrificing the quality of detection results. YOLO introduced anchor-based from YOLOv2 to improve the precision of boundary box prediction. However, from the YOLOX version to the latest YOLOv9, the anchor-free method has been used. The next section of this paper will introduce the anchor-free algorithm in detail.

#### 4.2.3 Anchor-free target detection algorithm

**Figure 10:**
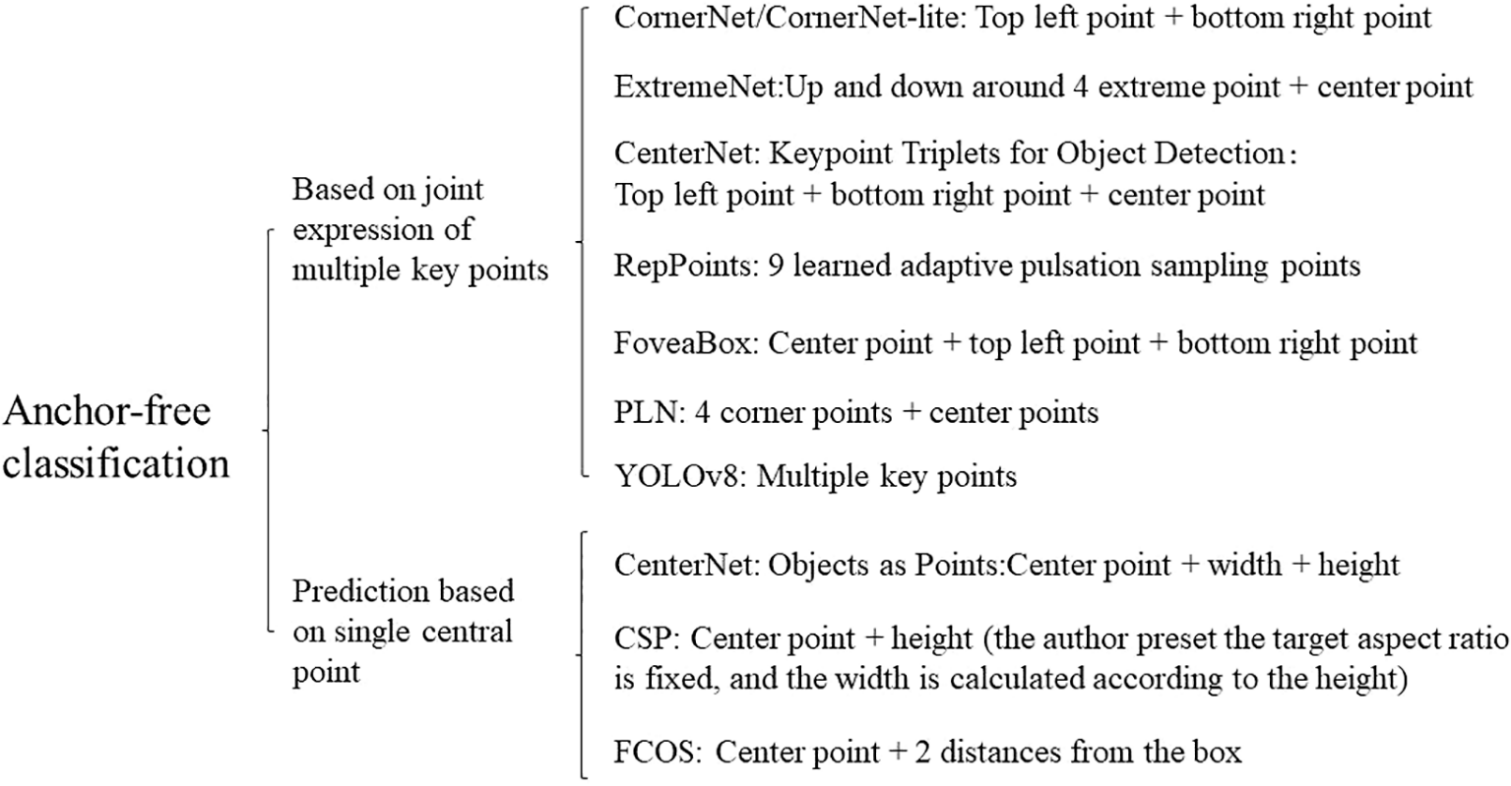
Classification of anchor-free detector.

The mainstream algorithms of the target detection model are mostly Anchor-based detection algorithms. This type of algorithm uses anchor boxes of different sizes and shapes to regression and classify the targets, which can directly classify targets and bounding box regression, with better detection effect, especially for small target detection has significant improvement, but it still has the following shortcomings: the anchor frame parameter design is more complex, and it needs to set a lot of artificial hyper-parameters, such as the size, length, width, etc., these parameters will affect the detection performance of the detector; The design scale and shape of the anchor frame detector are redundant, resulting in more negative samples, which makes the positive and negative samples unbalanced. A large number of redundant anchor frames will also increase the calculation cost. In response to these problems, anchor-free target detection algorithms are beginning to emerge. Anchor-free detection algorithms are a kind of target detection method. Different from the traditional method using predefined anchor frames, it does not need predefined anchor frames but directly predicts the location and category of the targets through the network. This method divides the recognition into two sub-problems of determining the object center and predicting the four borders, it only needs to regress the target center point, width, and height, which reduces the time-consuming and computing power and can be more adaptive to targets of different sizes and shapes. However, since anchor frames are not used and the anchor-free detection algorithm predicts only one frame at each position, some algorithms may have poor detection effects in some scenarios, such as overlapping or occlusion scenarios with a leakage detection problem. Anchor-free detection methods can be divided into two main categories based on single central point prediction and multi-key point joint expression (Zhang et al., 2022a), as shown in **Figure 10**.

The pixels on a feature map are called Anchor Points, which in target detection are also called Anchor frames, and are predefined frames used to generate candidate target frames. The prediction method based on a single central point is called Anchor Point Detector, and the role of anchor points in target detection algorithms is to be able to capture the target in the image at different scales and aspect ratios. The anchor point detector encodes the real frame as the anchor points and its positions are associated with the features. CenterNet, FCOS, and CSP are the representative algorithms of the anchor point detector. The anchor detection methods are mainly concerned with locating the position of the target and bounding frame in the image. Anchor points are usually placed at every location in the image, and each anchor point has a different size and aspect ratio. The object detection algorithm applies these anchor points to each location in the image to generate a series of candidate target frames that can cover targets of different sizes and shapes. The key point detector decodes the key points into the prediction frame by predicting the location of key points such as corner point, center point, or Extreme point in the bounding frame, CornerNet, ExtremeNe, etc. are its representative algorithms. This method focuses on detecting specific key points or feature points of the object, rather than predicting the bounding frame directly. Key points are usually points on the object with significant properties, and by detecting these points, the position and attitude of the object can be inferred. The fruit target recognition research results of some anchor-free target detection algorithms are shown in **Table 4**. To sum up, due to the shortcomings of the anchor-based algorithm, relevant scholars have proposed an anchor-free algorithm to address these shortcomings. From the existing research, it can be seen that the anchor-free algorithm performs better than anchor-based algorithms in certain scenarios. However, due to the relatively late emergence of anchor-free algorithms and short research time, many algorithms are currently not suitable for general target detection. The target recognition anchor-based algorithm is still in the mainstream in terms of application. Compared with anchor-based algorithms, this kind of algorithm has the advantages of strong robustness, short training time, and can avoid sample imbalance problems during the training process, this type of algorithm itself has not encountered a research bottleneck and is still in the rapid development stage, it will still be one of the research hotspots of target detection algorithms in the next few years.

**Table 4:** Research results of fruit target recognition based on anchor-free detection algorithms.

Recognition algorithm	Application scenarios	Technical principles and characteristics	Identification effect and evaluation index	Research scholars
FCOS	Natural environment, apples	Backbone network used DarkNet19, which improved loss function: fusion union intersection ratio and focus loss	The error caused by the imbalance of positive and negative sample ratio was reduced, precision was 96.00%, mAP was 96.30%	(Long et al., 2021b)
	Same color, light, occlusion, green apple	New detection method: the RFPN structure was introduced to replace FPN, and the two-layer convolutional attention network was added	The detection precision was 81.20%, the segmentation precision was 85.3%, model size was 39.7MB	(Liu et al., 2022b)
	Coloring, occlusion, light, green fruit	Add LSC module, add deformable convolution, FPN cross-connect, add attention mechanism in size, space, and channel	The model size was 38.65MB, average precision was 63.0%(green apple), 75.2%(green persimmon)	(Zhao et al., 2023)
	Light, shade, coloration, green apple	The feature extraction capability of CNN was integrated, and a bottom-up feature fusion architecture was added	The average precision was 85.6%, model size was 32.0MB	(Zhong-hua et al., 2022)
FoveaBox	Same color, green apple	Fast-FDM model was proposed, EfficientNetV2-S was used for the backbone network, BiFPN was used for feature extraction	The mAP for detecting green apples was 62.3%	(Jia et al., 2022a)
CenterNet	Apple	Improved original network Design Lightweight network Tiny Hourglass-24 backbone Network	In dense scenes, the mAP was 93.63%, F1 was 92.91.00%, recognition time was 69 ms/graph	(Yang et al., 2022a)
	Apple	Adopt the improved MobileNetv3 as CenterNet’s backbone network	The mAP was 88.90%, the model size was 14.2MB, detection speed was 8.1 f/s	(Xue et al., 2020)
YOLOX - S	Kiwi fruit	Lightweight, multi-scale feature set, improved activation function and loss function	The precision was 6.52% improved, the model parameters was 44.8% reduced, detection speed was 63.90% improved	(Zhou et al., 2022)
YOLOX - ViT	Small target, occlusion, overlap, tomato	Fruit and flower collaborative recognition method, image combination enhancement, and front-end ViT classification network are introduced	The mAP was 92.30%, detection speed was 28.46 f/s	(Lv et al., 2023)
YOLOX - Tiny	Natural environment, apple	A lightweight Shufflenetv2YOLOX detection method is proposed, and CBAM attention and ASFF feature fusion modules are added	The mAP was 96.76%, precision was 95.62%, recall was 93.75%, F1 was 95.00% and speed was 65 f/s	(Ji et al., 2022)
	Natural environment, apple	Proposed lightweight Lad-YXNet model, introduced ECA and SA lightweight attention modules, and built SDCLayer modules	The average precision was 94.88%, the detection time was 10.06 ms/graph, and the model size was 16.6MB	(Hu et al., 2022)

### 4.3 Target segmentation method based on deep learning

#### 4.3.1 Semantic segmentation model based on deep learning

The semantic segmentation model based on deep learning aims to assign each pixel in the image to the corresponding semantic category, to achieve pixel-level image segmentation, which is a more advanced task of target detection. Classifying each pixel point in the target image is the purpose of semantic segmentation. The following are some common semantic segmentation models based on deep learning:

Fully Convolutional Network (FCN) (Long et al., 2015): FCN is the pioneering work of the target detection algorithm in the field of semantic segmentation, released in 2014. FCN is a model that extends the traditional CNN into a full convolutional structure with the core idea of feature fusion. It applies CNN to semantic segmentation tasks by restoring resolution through layer-by-layer up-sampling. The biggest feature is that FCN can retain both the location information and semantic information of the target, and can classify the target at the pixel level to complete the task of target segmentation.

U-Net (Ronneberger et al., 2015): Released in 2015, the core idea of U-Net is the stitching of feature maps, which are widely used in semantic segmentation tasks. Its structure includes two parts: encoder (under-sampling) and decoder (up-sampling) and achieves fine image segmentation through a series of convolutional and up-sampling layers.

SegNet (Badrinarayanan et al., 2017): SegNet was released in 2015, the core idea is to put forward the max pool index to up-sampling, its backbone network is two VGG16 removed the full connection layer, forming an encoder-decoder structure for image segmentation, the encoder extract features, the decoder gradually restore resolution.

DeepLab Series: DeepLab is a series of models that use dilated convolution to expand the receptive field, thereby integrating contextual information while maintaining the resolution. DeepLab v1 (Chen et al., 2014), published in 2014, is an improved full-convolutional layer network based on VGG-16. The core idea is to use spatial convolution to expand the receptive field and conditionally refine the boundary randomly. DeepLab v2 (Chen et al., 2017), published in 2016, uses ResNet-101 and VGG16 models as the base network. The main difference from DeepLab v1 is the introduction of atrous spatial pyramid pooling (ASPP) structure with hollow convolution, which improved the segmentation precision. DeepLab v3 (Chen et al., 2018) uses ResNet-101 and Xception as backbone networks respectively, and introduces deep separable convolution to ASPP structures, effectively reducing the computational complexity of the model while maintaining the performance. DeepLab v3+ adds a decoder module based on DeepLab v3 and uses Aligned Xception as the backbone network.

PSPNet (Zhao et al., 2017) (Pyramid Scene Parsing Network): PSPNet was released in 2017, the core idea is to propose the Pyramid pooling module, which captures context information of different scales through pyramid pooling layers, and improves the performance of semantic segmentation.

ENet (Paszke et al., 2016) (efficient neural network): ENet is a lightweight semantic segmentation model designed for real-time performance, suitable for embedded and mobile devices.

#### 4.3.2 Instance segmentation model based on deep learning

Assigning semantic labels and instance labels to all pixels to segment target instances is called instance segmentation. The instance segmentation model based on deep learning aims to segment each target instance in the image into separate parts, and each instance is assigned a unique tag, i.e., a pixel-level segmentation mask is assigned to each target. Compared with semantic segmentation, it can provide more detailed image information such as the location and number of detected objects. Mask R - CNN is the most representative algorithm for fruit target instance segmentation. Published in 2017, Mask R - CNN (He et al., 2017a) extends the target detection model Faster R - CNN and simultaneously predicts the segmentation mask of the target category, bounding frame, and pixel level. **Table 5** lists the research achievements of some scholars using a segmentation algorithm based on deep learning for fruit target recognition. In **Table 5**, many scholars have used different target segmentation methods to solve the problem of fruit recognition in different scenarios and achieved good recognition results. However, target segmentation is to detect all targets in the image, and solve the problem of which object or scene each pixel belongs to at the pixel level. The question of which target or scene it belongs to has high computational cost and complexity, and the overall detection and segmentation take a long time, which is not conducive to real-time picking in orchards. To improve the overall detection speed of the network model, a large number of scholars have begun to conduct lightweight research on the model to improve the detection speed of the target recognition network model. The next section will introduce the lightweight method of the network model in detail.

**Table 5:** Research results of fruit target recognition based on deep learning segmentation algorithm.

Recognition algorithm	Application scenarios	Technical principles and characteristics	Identification effect and evaluation index	Research scholars
Mask R – CNN	Occlusion, Apple	Mask R - CNN combined with SfM photogrammetry technology to generate a 3D point cloud to achieve apple fruit segmentation	The mAP was 85.99%, F1 was 86.00%, high occlusion and small target, there was missegmentation	(Gené-Mola et al., 2020)
	Grapes	Segmentation of grape clusters in natural scenes based on Mask R - CNN	The precision rate was 92.00%, recall rate was 86.00%, F1 value was 88.90%	(Santos et al., 2020)
	Strawberries	The backbone network uses Resnet50 combined with FPN for feature extraction	The precision rate was 95.78%, the recall rate was 95.41%, average MIoU was 89.85%	(Yu et al., 2019)
	Many fruit, occlusion, citrus	A segmented labeling method for random and irregular branches was proposed, and a segmented merging algorithm was used	The average precision of fruit was 88.15%, the recall rate was 79.85%, average precision of branch was 96.27%	(Yang et al., 2020)
	Tomato	Swin Small + Cascade Mask R - CNN network model was applied for detection and semantic segmentation	When IoU takes 0, 0.5, and 0.75, the mask AP increases by 7.8, 6.4 and 7.2 percentage points respectively	(Zhang M. et al., 2022)
	Cherry tomatoes	Improvement: RGB and depth image dual-module data fusion, using multi-class prediction subnetwork	The precision rate was 93.76% (11.53% improved), recall rate was 94.47%(11.53% improved)	(Xu et al., 2022)
	Tomato	A multi-source information fusion method of RGB image, depth image, and infrared image is proposed	The precision rate was 98.30%, IOU was 91.6%	(Wang et al., 2021c)
	Cherry tomatoes	Proposed Fuzzy Mask R - CNN model	The precision was 98.00%, the overall weighted precision was 96.14%, recall rate was 95.91%	(Huang et al., 2020)
	Apple	A new method of binocular localization based on segmentation neural network was proposed	The IoU was 80.11%(detection), IoU was 84.39%(segmentation), precision was 99.49%	(Zhang et al., 2023)
	Occlusion, overlap, grape	The backbone network ResNet50-FPN-ED was proposed, the ECA mechanism was introduced, and DUC was used for feature fusion	The average precision of instance segmentation was 59.5%. AP was 1.6% better than the original Mask R -CNN	(Shen et al., 2022)
DasNet - v2	Light, Occlusion, Apple	Improved: Lightweight, adds instance split branches to FPN, simplifies FPN, and adopts encoder with cavity convolution	The backbone network was ResNet-101 with a recall rate of 86.80%, precision rate of 88.00%, and segmentation precision rate of 87.30%	(Kang and Chen, 2020)
U-Net	Dragon fruit	Introduction of SCSE attention mechanism and integration into residual module DRB	The mIoU was 86.69%, mPA was 93.89%, average error of 3D attitude estimation was 8.8°	(Lixue et al., 2023)
Deeplab+ResNet	Apple	Three architectures were compared:Deeplab v3 + ResNet-18, VGG-16 and VGG-19	ResNet-18 had 97% mAP and IoU of 0.69, both of which were better than VGG networks	(Zhang et al., 2021b)
CSP-ResNet50	different ripeness, tomato	Fusing the interstage local network CSPNet with the original residual network ResNet	The mean precision was 95.45%, was F1 91.2%, segmentation time 0.658/graph	(Long et al., 2021a)
PSPNet	Different poses, dragon fruit	A method for detecting dragon fruit endpoints based on PSPNet was proposed	The precision was 84.4%, the recall rate was 92.4%, average precision was 93.2%	(Zhou et al., 2023a)
	Litchi	YOLOv5 and PSPNet were used as the main stem detection and segmentation model of litchi	The recall and precision were 76.29% and 92.50%, respectively	(Qi et al., 2022)
	Natural environment	Embedding CBAM attention module and improving semantic segmentation model; Fusion of multiple feature layers	IoU and mAP were 87.42% and 95.73% respectively, which were 4.36% and 9.95% higher than the origirepiPSPNet model	(Chen et al., 2021b)
Deeplab	Rotten fruit, apple	DeepMDSCBA segmentation model was proposed, and feature extraction used MobileNet, depth convolution, and attention module	IoU and mAP were 87.42% and 95.73% respectively, which were 4.36% and 9.95% higher than the original PSPNet model	(Mo et al., 2022)
	Litchi	DeepLabV3+ fusion anomaly depth separable convolution feature; Encoding, decoded structure, and space pyramid pool were adopted	MIoU was 76.5% (14.4% improved), with stronger robustness	(Peng et al., 2020)
	Banana	The CNN Deeplab V3+ model was combined with the classical image processing algorithm	The target segmentation MIoU was 87.8%, the average precision was 93.6%, detection precision was 86%	(Wu et al., 2023)

### 4.4 Fruit target recognition method based on network compression and acceleration

With the development of computer hardware and the enhancement of GPU processing power, the computing power foundation has been provided for the application of target recognition algorithms. For fruit recognition with multiple interference factors under complex orchard background, to meet more recognition requirements and higher recognition precision, the neural network model of target recognition has gradually become more and more complex from the initial simple structure, with deeper and deeper model depths, and the model parameters are also increasing, resulting in the explosive growth of model size and calculation cost, larger memory storage and growing number of floating point calculation increase the training cost and calculation time, bringing new challenges to the deployment of the model on embedded devices (Zhu et al., 2021; Wang et al., 2023a). Therefore, how to carry out model compression and acceleration to achieve model lightweight without affecting the performance of deep learning models has become a research hotspot.

Current lightweight fruit target recognition models aim to achieve efficient fruit target recognition while maintaining low computing resources and memory consumption. The optimization mainly focused on reducing the computation amount and model parameters, reducing the actual running time, simplifying the underlying implementation, and simplifying the model structure. Model compression and acceleration are the main methods and means to achieve model lightweight, generally through the simplification of neural network parameter redundancy and network structure redundancy to achieve not only not affect the completion of the recognition tasks, but also to obtain a network model with fewer parameters and more streamlined structure.

The methods of model compression and acceleration can be divided into three types: compression parameter, compression structure, and hybrid compression, in which the compression parameter can be subdivided into strategies such as parameter pruning, parameter quantization, low-rank decomposition, and parameter sharing. The purpose of parameter pruning is to reduce the number of parameters in the model. By designing evaluation criteria on the importance of parameters, eliminating unnecessary connections or layers in the model to reduce parameters; The essence of parameter quantization is to quantize network parameters (weights) and convert floating point digits to reduce storage space; Low-rank decomposition refers to the reduction of high-dimensional parameter vectors to sparse low-dimensional vectors; The purpose of parameter sharing is to mapping the internal parameters of the network by using methods such as structural matrix or clustering, to achieve the sharing of parameters in different layers and to reduce redundant storage and training time.

**Figure 11:**
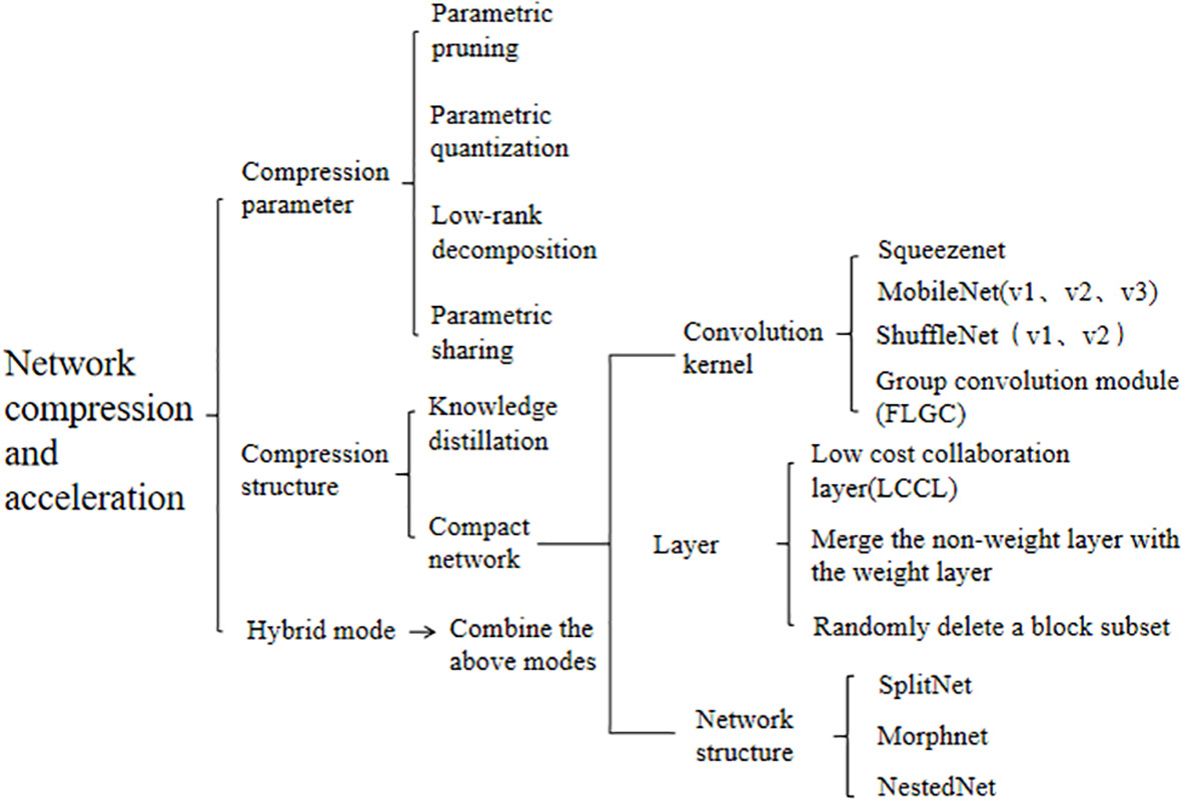
Common methods of network compression and acceleration.

The compressed structure can be divided into strategies such as knowledge distillation and compact network. Knowledge distillation refers to distilling small models from large models to maintain performance and reduce parameters; Compact networks refer to designing new networks in terms of convolution kernels, special layers, and network structure, reducing the computation by designing fewer channels, smaller convolution kernels, using deeply separable convolutions, lightweight modules, simple network structure (SqueezeNet, MobileNet, etc.), and optimizing network connections and hierarchical structure to reduce the number of parameters, reduce the computational complexity and extract features efficiently; The compact structure design directly optimizes the model from the perspective of model structure, compared with the model compression, the compact structure design has a more obvious effect in model acceleration and can reduce the number of parameters and calculations amount of the model to a greater extent, and improve the detection speed of the model. Therefore, the lightweight model design of a compact network is the main development direction of the target detection algorithms used in embedded device transplantation and mobile terminals in the future. **Figure 11** lists the compression acceleration networks that have performed well in recent years. **Table 6** lists some research results of fruit target recognition based on network compression and acceleration models.

**Table 6:** Research results of fruit target recognition based on network compression and acceleration model.

Recognition algorithm	Application scenarios	Technical principles and characteristics	Identification effect and evaluation index	Research scholars
MobileNet	Drone picking, longan	MobileNet was adopted to improve the original backbone network of the YOLOv4 model	The mAP was 89.73%(8.01% improved), the average detection time was 68 ms, model size was 46.5 MB	(Li et al., 2021a)
MobileNetv2	Small target, grapes	MobileNetv2 was adopted to replace the original backbone network of YOLOv3, M - Res2Net module was introduced; Improved loss function	The average precision was 81.20%, the average detection time was 6.29 ms/graph, model size was 44 MB	(Li et al., 2021b)
MobileNetv3	Multiple types of fruit	An end-to-end detection model was designed based on MobileNetV3 network architecture	The precision was 93.64%, the detection time was 8.4 ms/graph	(Cao et al., 2022)
	Dragon Fruit	MobileNetv3 was adopted to replace the original backbone network of YOLOv4, and upsampled feature fusion was added	The AP was 96.48%, recall rate was 95.00%, mIOU was 81.09%, model size was 2.7 MB	(Jinpeng et al., 2020)
	Dense fruit, occlusion, Cherry tomatoes	YOLOv4-LITE lightweight detection model was proposed, and MobileNet-v3 was used for feature extraction; Depthseparable convolution	The model size was 45.3MB, detection time was 3.01 ms/graph, mAP was 99.74%, precision was 99.15%	(Zhang et al., 2021a)
	Complex Network, apple	Replace the YOLOv4 backbone network with MobileNetv3 and introduce a coordinated attention mechanism	The AP was 92.23%, the model size was 54.1 MB, detection speed on the embedded platform was 15.11 f/s	(Wang et al., 2022)
Squeezenet	Mango	Feature extraction of the SqueezeNet model was visually analyzed, redundant layers were removed, and the convolution kernel was modified	The model size was 0.87MB, the computation amount was 181 MFLOPS, average precision was 95.64%	(Wei et al., 2022)
ShuffleNetV2	Light, occlusion, shadow, jujube	ShuffleNet V2 was adopted to improve the yolov5 backbone network, and the data loading module Stem was proposed, PANet was replaced by BiFPN	The number and size of model parameters were 6.25% and 8.33% of that of the original network, respectively. Precision, Recall, F1-score, AP, and FPS were all improved.	(Qiao et al., 2022)
YOLOX-s	Occlusion, apple	A groundbreaking multi-type occlusion Apple datasets design and data balance enhancement method was proposed	Precision increased from 0.894 to 0.974, recall rate from 0.845 to 0.972, mAP0.5 from 0.982 to 0.919	(Li et al., 2022b)
YOLOX	Occlusion, small target, Cherry Tomatoes	The YOLOX-Dense-CT model with the DenseNet backbone network was proposed, and the CBAM attention mechanism was adopted	The mAP was 94.80% (up 4.02%), model size was 34.6MB (down 19.6MB)	(Zheng et al., 2022a)
MFN	Same color, banana	The banana stem segmentation method is based on lightweight multifeature fusion Deep neural Network (MFN)	The number of model parameters was reduced, the operation efficiency was improved, and the model can be transplanted to mobile devices	(Chen et al., 2021c)
GhostNet	Occlusion, light, small target, Apple	An improved yolov4 network was proposed. The neck and YOLO head structures can be reconstructed by introducing depth	The mAP was 95.72%(3.45% improved), the network size was 37.9MB, and speed was increased by 5.7 FPS	(Zhang C. et al., 2022)

The above systematically describes the process and classification of target recognition methods based on deep learning and the research results of many scholars in the related algorithms. In general, compared with the single-stage recognition algorithms, the two-stage recognition algorithms can obtain higher recognition precision and have better performance in large targets and complex scenarios, but the recognition speed is slow; The single-stage algorithms have a faster detection speed, but it is easy to produce a higher false detection rate in small target detection and more complex environments; Compared with anchor-based target detection algorithm, the anchor-free target detection algorithm has stronger generalization ability, more concise framework, and high precision of abnormal scale target detection, which reduces the time and computing power. However, in some scenarios (occlusion, overlap, etc.), there will be a leakage detection phenomenon. For multiscale target detection and small target detection, the precision is lower than that of the anchor-based detection algorithm. Semantic segmentation is the advanced task of image detection, which is used to judge which target the pixels in the image belong to. Instance segmentation can be regarded as an advanced task that unifies target detection and semantic segmentation. The advantage is that the bounding box instance segmentation of contrast target detection can be accurate to the edge of the object, while the same target attribute instance segmentation of contrast semantic segmentation needs to label different individuals of the same target on the graph. The lightweight network based on network compression and acceleration is designed to achieve efficient fruit target recognition while maintaining low computing resources and memory consumption, which is also one of the current research hotspots in orchard target recognition.

In general, with the rapid development of deep learning, the application of fruit target recognition methods based on deep learning in orchard fruit recognition tasks in recent years far exceeds the application of traditional fruit target recognition methods. The single-stage target detection algorithm has the advantages of detection speed and the anchor-free recognition algorithm has the advantages of better generalization ability and lower computing power consumption, which is more suitable for the orchard picking target recognition task. If you are a beginner and want to achieve real-time detection tasks, the YOLO series algorithm is a good choice, which is an end-to-end single-stage detection algorithm. The latest version of YOLO adopts the principle of anchor-free detection, and many scholars are still continuously improving YOLO from the perspective of network model compression and acceleration.

## 5 Conclusion and future perspectives

As mentioned above, although the relatively mature target recognition network model based on deep learning has been widely used in various fruit recognition tasks, most of the researches on network models are based on the original model structure, aiming at specific recognition scenarios, by changing the model structure, adding attention mechanism or using transfer learning to improve the detection performance of the model. Although certain results can be achieved, as mentioned above, each model still has different degrees of shortcomings that make it difficult to completely solve the interference problem caused by the complex orchard environment to the target recognition task, and there are still many problems and challenges in the actual application of the model to the fruit picking robot. Be specifically manifested in

1. It is more difficult to prepare large-scale public standard datasets for orchards. At present, the research results of different scholars are only based on small-scale datasets prepared by individuals, which cannot fully reflect the performance of research algorithms. The fruit target datasets should contain all the interference conditions such as shadows, occlusion, fruit overlap, night environment, uneven illumination, and the same color scheme in the complex orchard environment, and fruit agricultural products have a certain growth cycle, the data collection will be affected by many uncontrollable factors such as weather and region, and the data processing will also be affected by human factors. Therefore, the preparation of large-scale and high-quality public orchard datasets is one of the difficulties in fruit target recognition tasks.
2. The detection model recognition algorithms have some limitations. Although deep learning-based CNN has shown good performance in fruit target recognition, it can be seen from the above that due to the complexity and non-structure of the natural working environment and the uncertainty of the growth state of fruit, all kinds of network models have varying degrees of shortcomings. At present, most mature target recognition models online are supervised learning models. To cope with the influence of various interference factors in complex orchard conditions, the model needs to introduce more network structure layers, which leads to more complex models, increases the calculation time, reduces the real-time performance of the system, and affects the picking efficiency.
3. The algorithms are not universal. Most of the deep learning recognition algorithms are supervised learning models, which cannot automatically adapt to the variability of the natural environment in the orchard and the growth differences between different fruits, are limited to specific picking environment and picking objects, and rely too much on the label information of datasets. For specific picking objects, corresponding ripe fruit datasets need to be made for target recognition training. It is necessary to re-prepare and train the datasets when the target fruits are replaced, which restricts the popularization and application of the vision system. The development of a fruit target recognition model with high versatility is conducive to improving the universality of picking robots.
4. For the overlapping and complex occlusion of fruits, although many scholars have carried out relevant research, effective solutions have not yet been obtained.
5. The stability, generalization, and robustness of the model in complex scenarios are poor. The interference factors in the orchard’s natural environment have the characteristics of randomness and uncertainty, which will affect the recognition results. The model can only with high stability, generalization, and robustness to have a better detection effect under the influence of the interference factors in the natural environment of the orchard. Therefore, how to improve the performance of the model in complex scenes of orchards is currently a difficult problem in the field of fruit picking target recognition.

Given the above problems, future research on orchard target recognition should focus on the following aspects

1. Investigate weakly supervised or unsupervised deep learning models (or find an alternative to manually labeling samples). The limited sample data is used to effectively train the model, reduce the number of label data, reduce labor costs, and improve the flexibility of detection and learning efficiency.
2. Compression and acceleration of deep neural networks. On the premise of ensuring the model detection effect, the models are compressed and accelerated to obtain a lightweight network with a compact structure, fewer parameters, and higher computing power, improve the detection speed of lightweight models, and create conditions for the deployment of models on embedded devices with limited computing power. The development of models that can be used for real-time and accurate detection of fruit targets by edge devices is one of the research hotspots in future fruit target recognition.
3. In the future, it may be more inclined to anchor-free detection algorithms, with the research being more focused on the accurate recognition of small targets, occlusions, and dense fruits. Compared with an anchor-based algorithm, the precision is poor, but it reduces time-consuming and computing power, has faster detection speed, and can be more adaptive to targets of different sizes and shapes, which is more suitable for real-time orchard-picking tasks. However, for fruit overlap and occlusion, which is the difficulty of the orchard recognition task, the anchor-free algorithm has the problem of false detection at present, and there is still a lot of room for improvement.
4. Improve the visual working environment and integrate the recognition algorithm with the picking strategy. The complexity and non-structure of the natural working environment of the orchards is one of the main reasons for the difficulty of fruit target recognition at present. It is possible to change the planting mode to build standardized orchards, such as horizontal trellis-type planting patterns, Y-type planting patterns, trunk-type planting patterns, etc. Then, corresponding picking strategies can be formulated according to different planting patterns to artificially reduce the phenomenon of branches and leaves occlusion and fruit overlap. So that the difficulty of target recognition is reduced, and the precision, universality, and real-time performance of the recognition algorithm are improved effectively.
5. Improve the robustness and generalization of the algorithm, and introduce a new algorithm that is more suitable for orchard fruit recognition tasks. According to the characteristics of the actual working environment of orchards and the uncertainty of influencing factors, the advantages of various current target recognition algorithms should be integrated to further improve the fruit target recognition algorithm, to overcome the recognition errors caused by the randomness of environmental factors, to ensure the robustness and generalization of the network model, and to introduce recognition algorithms more suitable for the natural environment of orchards.

## Data Availability

The original contributions presented in the study are included in the article/supplementary material. Further inquiries can be directed to the corresponding author.
